# Representational structure or task structure? Bias in neural representational similarity analysis and a Bayesian method for reducing bias

**DOI:** 10.1371/journal.pcbi.1006299

**Published:** 2019-05-24

**Authors:** Ming Bo Cai, Nicolas W. Schuck, Jonathan W. Pillow, Yael Niv

**Affiliations:** 1 Princeton Neuroscience Institute, Princeton University, Princeton, New Jersey, United States of America; 2 Max Planck Research Group Neurocode, Max Planck Institute for Human Development, Berlin, Germany; 3 Max Planck UCL Centre for Computational Psychiatry and Ageing Research, Berlin, Germany; 4 Department of Psychology, Princeton University, Princeton, New Jersey, United States of America; Oxford University, UNITED KINGDOM

## Abstract

The activity of neural populations in the brains of humans and animals can exhibit vastly different spatial patterns when faced with different tasks or environmental stimuli. The degrees of similarity between these neural activity patterns in response to different events are used to characterize the representational structure of cognitive states in a neural population. The dominant methods of investigating this similarity structure first estimate neural activity patterns from noisy neural imaging data using linear regression, and then examine the similarity between the estimated patterns. Here, we show that this approach introduces spurious bias structure in the resulting similarity matrix, in particular when applied to fMRI data. This problem is especially severe when the signal-to-noise ratio is low and in cases where experimental conditions cannot be fully randomized in a task. We propose Bayesian Representational Similarity Analysis (BRSA), an alternative method for computing representational similarity, in which we treat the covariance structure of neural activity patterns as a hyper-parameter in a generative model of the neural data. By marginalizing over the unknown activity patterns, we can directly estimate this covariance structure from imaging data. This method offers significant reductions in bias and allows estimation of neural representational similarity with previously unattained levels of precision at low signal-to-noise ratio, without losing the possibility of deriving an interpretable distance measure from the estimated similarity. The method is closely related to Pattern Component Model (PCM), but instead of modeling the estimated neural patterns as in PCM, BRSA models the imaging data directly and is suited for analyzing data in which the order of task conditions is not fully counterbalanced. The probabilistic framework allows for jointly analyzing data from a group of participants. The method can also simultaneously estimate a signal-to-noise ratio map that shows where the learned representational structure is supported more strongly. Both this map and the learned covariance matrix can be used as a structured prior for maximum *a posteriori* estimation of neural activity patterns, which can be further used for fMRI decoding. Our method therefore paves the way towards a more unified and principled analysis of neural representations underlying fMRI signals. We make our tool freely available in Brain Imaging Analysis Kit (BrainIAK).

## Author summary

We show the severity of the bias introduced when performing representational similarity analysis (RSA) based on neural activity pattern estimated within imaging runs. Our Bayesian RSA method significantly reduces the bias and can learn a shared representational structure across multiple participants. We also demonstrate its extension as a new multi-class decoding tool.

## Introduction

Functional magnetic resonance imaging (fMRI) measures the blood-oxygen-level-dependent (BOLD) signals [1], which rise to peak ∼6 seconds after neuronal activity increases in a local region [2]. Because of its non-invasiveness, full-brain coverage, and relatively favorable trade-off between spatial and temporal resolution, fMRI has been a powerful tool to study the neural correlates of cognition [3–5]. In the last decade, research has moved beyond simply localizing the brain regions selectively activated by cognitive processes and the focus has been increasingly placed on the relationship between the detailed spatial patterns of neural activity and cognitive processes [6, 7].

An important tool for characterizing the functional architecture of the brain is representational similarity analysis (RSA) [8]. This classic method first estimates the neural activity patterns from fMRI data recorded as participants observe a set of stimuli or experience a set of task conditions, and then calculates the similarity (e.g., by Pearson correlation) between each pair of the estimated patterns. The rationale is that if two stimuli are represented with similar codes in a brain region, the spatial patterns of neural activation in that region would be similar when processing these two stimuli. When using Pearson correlation as a similarity metric, the activity profile of each voxel to all the task conditions is essentially viewed as one independent sample from a multivariate normal distribution in a space spanned by the experimental conditions, which is characterized by its covariance matrix. Recently, it has been pointed out that RSA and two other approaches for understanding neural representational structure, namely encoding model [9] and pattern component modeling (PCM) [10], are closely related through the second moment statistics (the covariance matrix) of the true (unknown) activity patterns [11].

After the similarity matrix between all pairs of estimated activity patterns is calculated in a region of interest (ROI), it can be compared against similarity matrices predicted by candidate computational models. Researchers can also convert the similarity matrix into a representational dissimilarity matrix (RDM, e.g., 1 − *C*, for similarity *C* based on correlation) and visualize the structure of the representational space in the ROI by projecting the dissimilarity matrix to a low dimensional space [8]. Researchers might also test whether certain experimental manipulations change the degrees of similarity between neural patterns of interest [12, 13]. To list just a few applications from the field of visual neuroscience, RSA has revealed that humans and monkeys have highly similar representational structures in the inferotemporal (IT) cortex for images across various semantic categories [14]. It also revealed a continuum in the abstract representation of biological classes in human ventral object visual cortex [15] and that basic categorical structure gradually emerges through the hierarchy of visual cortex [16]. Because of the additional flexibility of exploring the structure of neural representation without building explicit computational models, RSA has also gained popularity among cognitive neuroscientists for studying more complex tasks beyond perception, such as decision making.

While RSA has been widely adopted in many fields of cognitive neuroscience, a few recent studies have revealed that the similarity structure estimated by standard RSA might be confounded by various factors. First, the calculated similarity between two neural patterns strongly depends on the time that elapsed between the two measured patterns: the closer the two patterns are in time, the more similar they are [17] [18]. Second, it was found that because different brain regions share some common time courses of fluctuation independent of the stimuli being presented (intrinsic fluctuations), RDMs between regions are highly similar when calculated based on patterns of the same trials of tasks but not when they are calculated based on separate trials (thus the intrinsic fluctuation are not shared across regions). This indicates that RSA can be strongly influenced by intrinsic fluctuation [17]. Lastly, Diedrichsen et al. (2011) pointed out that the noise in the estimated activity patterns can add a diagonal component to the condition-by-condition covariance matrix of the spatial patterns. This leads to over-estimation of the variance of the neural pattern and underestimation of correlation between true patterns, and this underestimation depends on signal-to-noise ratio in each ROI, making it difficult to make comparison of RDMs between regions [10].

Recognizing the first two issues, several groups have recently suggested modifications to RSA such as calculating similarity or distance between activity patterns estimated from separate fMRI runs [18, 19], henceforth referred to as cross-run RSA, and using a Taylor expansion to approximate and regress out the dependency of pattern similarity on the interval between events [18]. For the last issue, Diedrichsen et al. (2011) proposed PCM which models the condition-by-condition covariance matrix between estimated neural patterns as the sum of a diagonal component that reflects the contribution of noise in the estimated neural patterns to the covariance matrix and components reflecting the researcher’s hypothetical representational structure in the ROI [10]. These methods improve on traditional RSA, but are not explicitly directed at the source of the bias, and therefore only offer partial solutions.

Indeed, the severity of confounds in traditional RSA is not yet widely recognized. RSA based on neural patterns estimated within an imaging run is still commonly performed. Furthermore, sometimes a study might need to examine the representational similarity between task conditions within an imaging run, such that cross-run RSA is not feasible. The Taylor expansion approach to model the effect of event-interval can be difficult to set up when a task condition repeats several times in an experiment. There also lacks a detailed mathematical examination of the source of the bias and how different ways of applying RSA affect the bias. Researchers sometimes hold the view that RSA of raw fMRI patterns, instead of activity patterns (***β***) estimated through a general linear model (GLM) [20], does not suffer from the confounds mentioned above. Last but not least, the contribution of noise in the estimated neural patterns to the sample covariance matrix between patterns may not be restricted to the diagonal elements, as we will demonstrate below.

In this paper, we first compare the result of performing traditional RSA on a task-based fMRI dataset with the results obtained when performing the same analysis on white noise, to illustrate the severe bias and spurious similarity structure that can result from performing RSA on pattern estimates within imaging runs. By applying task-specific RSA on irrelevant resting-state fMRI data, we show that spurious structure also emerges when RSA is performed on the raw fMRI pattern rather than estimated task activation patterns. We observed that the spurious structure can be far from a diagonal matrix, and masks any true similarity structure. We then provide an analytic derivation to help understand the source of the bias in traditional RSA. Previously, we have proposed a method named Bayesian RSA (BRSA), which significantly reduced this bias and allows analysis within imaging runs [21]. BRSA is related to PCM in the sense that they both treat the true and unknown activity profiles of each voxel as a sample from a multivariate normal distribution and marginalize the true activity pattern in their analysis. The critical difference is that PCM models the estimated activity patterns of each trial or task condition, in which complex spurious correlation structure could have already been introduced during the estimation, while BRSA directly models the raw imaging data. Here, we further extend BRSA to explicitly model spatial noise correlation, thereby mitigating the second issue identified by Heriksson et al. [17], namely the intrinsic fluctuation not modelled by task events in an experiment. Furthermore, inspired by the methods of hyper-alignment [22] and shared response models [23], we extend our method to learn a shared representational similarity structure across multiple participants (Group BRSA) and demonstrate improved accuracy of this approach. Since our method significantly reduces bias in the estimated similarity matrix but does not fully eliminate it at regimes of very low signal-to-noise ratio (SNR), we further provide a cross-validation approach to detecting over-fitting to the data. Finally, we show that the learned representational structure can serve as an empirical prior to constrain the posterior estimation of activity patterns, which can be used to decode the cognitive state underlying activity observed in new fMRI data.

The algorithm in this paper is publicly available in the Python package Brain Imaging Analysis Kit (BrainIAK), under the *brainiak.reprsimil.brsa* module. Our previous version of Bayesian RSA method [21] with newly added modeling of spatial noise correlation is in the *BRSA* class of the module. The new version described in this paper is implemented in the *GBRSA* class and can be applied to either a single participant or a group of participants.

## Results

### Traditional RSA translates structured noise in estimated activity patterns into spurious similarity structure

Traditional RSA [8] first estimates the response amplitudes (*β*) of each voxel in an ROI to each task condition, and then calculates the similarity between the estimated spatial response patterns of that ROI to each pair of task conditions.

The estimation of *β* is based on a GLM. We denote the fMRI time series from an experiment as $Y\in R^{n_{T}\times n_{V}}$, with *n*_*T*_ being the number of time points and *n*_*V*_ the number of voxels. The GLM assumes that

Y=X·β+ϵ. (1)

$X\in R^{n_{T}\times n_{C}}$ is the “design matrix,” where *n*_*C*_ is the number of task conditions. Each column of the design matrix is constructed by convolving a hemodynamic response function (HRF) with a time series describing the onsets and duration of all events belonging to one task condition. The regressors composing the design matrix express the hypothesized response time course elicited by each task condition. Each voxel’s response amplitudes to different task conditions can differ. The response amplitudes of one voxel to all conditions forms that voxel’s response profile. All voxels’ response profiles form a matrix of spatial activity patterns $\beta \in R^{n_{C}\times n_{V}}$, with each row representing the spatial pattern of activity elicited by one task condition. The responses to all conditions are assumed to contribute linearly to the spatio-temporal fMRI signal through the temporal profile of hemodynamic response expressed in **X**. Thus, the measured **Y** is assumed to be a linear sum of **X** weighted by response amplitudes ***β***, corrupted by zero-mean noise ***ϵ***.

The goal of RSA is to understand the degree of similarity between each pair of spatial response patterns (i.e., between the rows of ***β***). But because the true ***β*** is not accessible, a point estimate of ***β***, derived through linear regression, is usually used as a surrogate:

$$\begin{array}{c} \hat{\beta }={\left(X^{T}X\right)}^{-1}X^{T}Y \end{array}$$ (2)

Similarity is then calculated between rows of $\hat{\beta }$. For instance, one measure of similarity that is frequently used is Pearson correlation. The similarity between patterns of condition ***i*** and ***j*** is assessed as

$$\begin{array}{c} C_{ij}=\frac{\left({\hat{\beta }}_{i}-\bar{{\hat{\beta }}_{i}}\right){\left({\hat{\beta }}_{i}-\bar{{\hat{\beta }}_{j}}\right)}^{T}}{n_{V}\sigma_{{\hat{\beta }}_{i}}\sigma_{{\hat{\beta }}_{j}}} \end{array}$$ (3)

where $\bar{{\hat{\beta }}_{i}}$ and $\sigma_{{\hat{\beta }}_{i}}$ are the mean and standard deviation of the estimated pattern of condition ***i*** across voxels.

**Fig 1:**
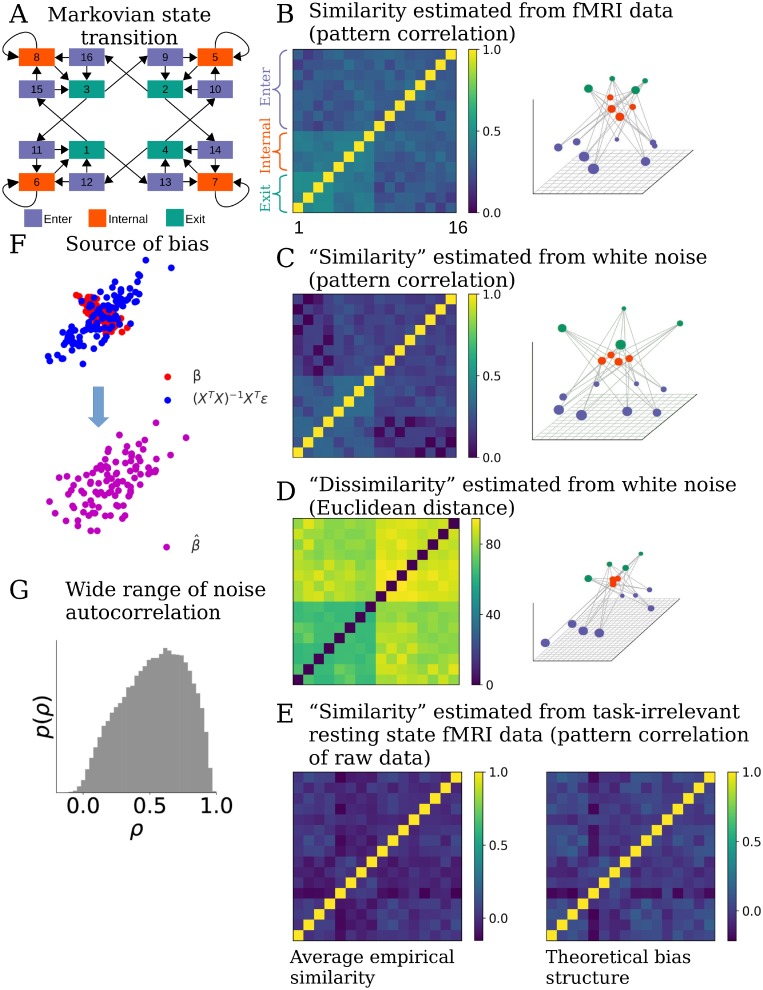
Standard RSA introduces bias structure to the similarity matrix. (**A**) A cognitive task including 16 different experimental conditions. Transitions between conditions follow a Markov process. Arrows indicate possible transitions, each with ***p*** = **0**.**5**. The task conditions can be grouped into 3 categories (color coded) according to their characteristic transition structure. (**B**) Standard RSA of activity patterns corresponding to each condition estimated from a brain region reveals a highly structured similarity matrix (left) that reflects aspects of the transition structure in the task. Converting the similarity matrix C to a distance matrix 1-C and projecting it to a low-dimensional space using MDS reveals a highly regular structure (right). Seeing such a result, one may infer that representational structure in the ROI strongly reflects the task structure. (**C**) However, applying RSA to regression estimates of of patterns obtained from pure white noise generates a very close similarity matrix (left), with a similar low-dimensional projection (right). This indicates that standard RSA can introduce spurious structure in the similarity matrix that does not exist in the data. (**D**) RSA Using Euclidean distance as a similarity metric applied to patterns estimated from the same noise (left) yields a slightly different, but still structured, similarity structure (right). (**E**) Calculating the correlation between raw patterns of resting state fMRI data (instead of patterns estimated by a GLM), assuming the same task structure as in (A), also generates spurious similarity structure, albeit different from those in (B-D). This structure is significantly correlated with the theoretical bias structure (details in main text). Left: average of the similarity structure based on raw patterns. Right: average of the theoretical bias similarity structure arising purely from task structure and fMRI noise autocorrelation. (**F**) The bias in this case comes from structured noise introduced during the GLM analysis. Assuming the true patterns ***β*** (red dots) of two task conditions are anti-correlated (the horizontal and vertical coordinates of each dot represent the response amplitudes of one voxel to the two task conditions), regression turns the noise ***ϵ*** in fMRI data into structured noise (**X**^***T***^ **X**)^−**1**^ **X**^***T***^***ϵ*** (blue dots). The correlation between the noises in the estimated patterns is often non-zero (assumed to be positive correlation here) due to the correlation structure in the design matrix and the autocorrelation property of the noise. The estimated patterns $\hat{\beta }$ (purple dots) are the sum of ***β*** and (**X**^***T***^ **X**)^−**1**^ **X**^***T***^***ϵ***. The correlation structure between estimated activity vectors for each condition will therefore differ from the correlation structure between the true patterns ***β***. (**G**) Distribution of the autocorrelation coefficients in a resting state fMRI dataset, estimated by fitting AR(1) model to the time series of each voxel resampled at TR = 2.4s. The wide range of degree of autocorrelation across voxels makes it difficulty to calculate a simple analytic form of the bias structure introduced by the structured noise, and calls for modeling the noise structure of each voxel separately.

To demonstrate the spurious structure that may appear in the result of traditional RSA, we first performed RSA on the fMRI data in one ROI, the orbitofrontal cortex, in a previous dataset involving a decision-making task [24]. The task included 16 different task conditions, or “states.” In each state, participants paid attention to one of two overlapping images (face or house) and made judgments about the image in the attended category. The transition between the 16 task states followed the Markov chain shown in Fig 1A, thus some states often preceded certain other states. The 16 states could be grouped into 3 categories according to the structure of transitions among states (the exact meaning of the states, or the 3 categories, are not important in the context of the discussion here.) We performed traditional RSA on the 16 estimated spatial response patterns corresponding to the 16 task states. To visualize the structure of the neural representation of the task states in the ROI, we used multi-dimensional scaling (MDS) [25] to project the 16-dimensional space defined by the distance (1—correlation) between states onto a 3-dimensional space (Fig 1B).

This projection appears to show clear grouping of the states in the orbitofrontal cortex consistent with the 3 categories, suggesting that this brain area represent this aspect of the task. However, a similar representational structure was also observed in other ROIs. In addition, when we applied the same GLM to randomly generated white noise and performed RSA on the resulting parameter estimates, the similarity matrix closely resembled the result found in the real fMRI data (Fig 1C). Since there is no task-related activity in the white noise, the structure obtained from white noise is clearly spurious and must reflect a bias introduced by the analysis. In fact, we found that the off-diagonal structure obtained from white noise (Fig 1C) explained **84** ± **12**% of the variance of the off-diagonals obtained from real data (Fig 1B). This shows that the bias introduced by traditional RSA can dominate the result, masking the real representational structure in the data.

To help understand this observation, we provide an analytic derivation of the bias with a few simplifying assumptions [21]. The calculation of the sample correlation of $\hat{\beta }$ in traditional RSA implies the implicit assumption that an underlying covariance structure exists that describes the distribution of ***β***, and the activity profile of each voxel is one sample from this distribution. Therefore, examining the relation between the covariance of $\hat{\beta }$ and that of true ***β*** will help us understand the bias in traditional RSA.

We assume that a covariance matrix **U** (of size ***n***_***C***_ × ***n***_***C***_) captures the true covariance structure of ***β*** across all voxels in the ROI: ***β*** ∼ **N**(**0**, **U**). Similarity measures such as correlation are derived from **U** by normalizing the diagonal elements to 1. It is well known that temporal autocorrelation exists in fMRI noise [26, 27]. To capture this, we assume that in each voxel ***ϵ*** ∼ ***N***(**0**, **Σ**_***ϵ***_), where $\Sigma_{\epsilon }\in R^{n_{T}\times n_{T}}$ is the temporal covariance of the noise (for illustration purposes, here we assume that all voxels have the same noise variance and autocorrelation, and temporarily assume the noise is spatially independent).

By substituting the expression for **Y** from Eq (1) into the point estimate of ***β*** (2), we obtain

$$\hat{\beta }={\left(X^{T}X\right)}^{-1}X^{T}X\beta +{\left(X^{T}X\right)}^{-1}X^{T}\epsilon =\beta +{\left(X^{T}X\right)}^{-1}X^{T}\epsilon$$ (4)

which means the point estimate of ***β*** is contaminated by a noise term (**X**^***T***^ **X**)^−**1**^ **X**^***T***^***ϵ***. Assuming that the signal ***β*** is independent from the noise ***ϵ***, it is then also independent from the linear transformation of the noise, (**X**^***T***^ **X**)^−**1**^ **X**^***T***^***ϵ***. Thus the covariance of $\hat{\beta }$ is the sum of the covariance of true ***β*** and the covariance of (**X**^***T***^ **X**)^−**1**^ **X**^***T***^***ϵ***:

$$\begin{array}{c} \hat{\beta }∼N(0,U+{\left(X^{T}X\right)}^{-1}X^{T}\Sigma_{\epsilon }X{\left(X^{T}X\right)}^{-1}) \end{array}$$ (5)

The term (**X**^***T***^ **X**)^−**1**^ **X**^***T***^***∑***_***ϵ***_ **X**(**X**^***T***^ **X**)^−**1**^ is the source of the bias in RSA. This bias originates from the structured noise (**X**^***T***^ **X**)^−**1**^ **X**^***T***^***ϵ*** in estimating $\hat{\beta }$. It depends on both the design matrix **X** and the temporal autocorrelation of the noise ***ϵ***. Fig 1F illustrates how structured noise can alter the correlation of noisy pattern estimates in a simple case of just two task conditions. Even if we assume the noise is temporally independent (i.e., **Σ**_***ϵ***_ is a diagonal matrix, which may be a valid assumption if one “pre-whitens” the data before further analysis [27]), the bias structure still exists but reduces to (**X**^***T***^ **X**)^−**1**^ ***σ***^**2**^, where ***σ***^**2**^ is the variance of the noise.

Since the covariance matrix of $\hat{\beta }$ is biased, its correlation is also distorted from the true correlation structure. This is because correlation is merely a rescaling of rows and columns of a covariance matrix. Fig 1C essentially illustrates this bias structure after being converted to correlation matrix (in this case, ***σ*** = 1 and ***β*** = **0**) as this RSA structure, by virture of being derived for white noise, can only result from structure in the design matrix **X**. In reality, both spatial and temporal correlations exist in fMRI noise, which complicates the structure of the bias. But the fact that bias in Fig 1C arises even when applying RSA to white noise which itself has no spatial-temporal correlation helps to emphasize the first contributor to the bias: the timing structure of the task, which is exhibited in the correlations between the regressors in the design matrix. Whenever the interval between events of two task conditions is shorter than the length of the HRF (which typically outlasts 12 s), correlation is introduced between their corresponding columns in the design matrix. The degree of correlation depends on the overlapping of the HRFs. If one task condition often closely precedes another, which is the case here as a consequence of the Markovian property of the task, their corresponding columns in the design matrix are more strongly correlated. As a result of these correlations, **X**^***T***^ **X** is not a diagonal matrix, and neither is its inverse (**X**^***T***^ **X**)^−**1**^.

In general, unless the order of task conditions is very well counterbalanced and randomized across participants, the noise (**X**^***T***^ **X**)^−**1**^ **X**^***T***^***ϵ*** in $\hat{\beta }$ is not i.i.d between task conditions. The bias term **B** = (**X**^***T***^ **X**)^−**1**^ **X**^***T***^ **Σ**_*ϵ*_ **X**(**X**^***T***^ **X**)^−**1**^ then deviates from a diagonal matrix and causes unequal distortion of the off-diagonal elements in the resulting correlation matrix of $\hat{\beta }$. These unequal distortions alter the order of ranking of the values of the off-diagonal elements. Therefore, rank correlation between the similarity matrix from traditional RSA and the similarity matrix of any candidate computational model is necessarily influenced by the bias. Conclusion based on such comparison between two similarity matrices or based on comparing a pair of off-diagonal elements within a neural similarity matrix becomes problematic, as long as the bias causes unequal distortion. Furthermore, if the design matrices also depend on participants’ performance such as errors and reaction time, the bias structure could depend on their performance as well. Comparison between neural representational structure and participants’ behavioral performance may also become problematic in such situations.

It is worth pointing out that the bias is not restricted to using correlation as metric of similarity. Because structured noise exists in $\hat{\beta }$, any distance metrics between rows of $\hat{\beta }$ estimated within imaging runs of fMRI data are likely biased. We can take Euclidean distance as an example. For any two task conditions *i* and *j*, the expectation of the distance between $\hat{\beta_{i}}$ and $\hat{\beta_{j}}$ is $\sum_{k=1}^{n_{V}}{\left(\beta_{ik}-\beta_{jk}\right)}^{2}+n_{V}\left(B_{ii}^{2}+B_{jj}^{2}-2B_{ij}^{2}\right)$, where **B** is the bias in the covariance structure. Therefore, the bias $n_{V}\left(B_{ii}^{2}+B_{jj}^{2}-2B_{ij}^{2}\right)$ in Euclidean distance also depends on the task timing structure and the property of noise. (See Fig 1D).

In our derivations above, point estimates of $\hat{\beta }$ introduce structured noise due to the correlation structure in the design matrix. One might think that the bias can be avoided if a design matrix is not used, i.e., if RSA is not performed after GLM analysis, but directly on the raw fMRI patterns. Such an approach still suffers from bias, for two reasons that we detail below.

First, RSA on the raw activity patterns suffers from the second contributor to the bias in RSA that comes from the temporal properties of fMRI noise. To understand this, consider that estimating activity pattern by averaging the raw patterns, for instance 6 sec after each event of a task condition (that is, at the approximate peak of the event-driven HRF) is equivalent to performing an alternative GLM analysis with a design matrix **X**_**6**_ that has delta functions 6 sec after each event. Although the columns of this design matrix **X**_**6**_ are orthogonal and ${\left(X_{6}^{T}X_{6}\right)}^{-1}$ becomes diagonal, the bias term is still not a diagonal matrix. Because of the autocorrelation structure **Σ**_***ϵ***_ in the noise, the bias term ${\left(X_{6}^{T}X_{6}\right)}^{-1}X_{6}^{T}\Sigma_{\epsilon }X_{6}{\left(X_{6}^{T}X_{6}\right)}^{-1}$ essentially becomes a sampling of the temporal covariance structure of noise at the distances of the inter-event intervals. In this way, timing structure of the task and autocorrelation of noise together still cause bias in the RSA result.

To illustrate this, we applied RSA to the raw patterns of an independent set of resting state fMRI data from the Human Connectome Project [28], pretending that the participants experienced events according to the 16-state task in Fig 1A. As shown in Fig 1E, even in the absence of any task-related signal spurious similarity structure emerges when RSA is applied to the raw patterns of resting state data. We then calculated the theoretical bias structure ${\left(X_{6}^{T}X_{6}\right)}^{-1}X_{6}^{T}\Sigma_{\epsilon }X_{6}{\left(X_{6}^{T}X_{6}\right)}^{-1}$ for each task sequence based on **X**_**6**_ of that sequence and **Σ**_***ϵ***_ estimated as the average noise temporal correlation matrix of the resting state data of three other participants (right figure of Fig 1E). The off-diagonal elements of all the similarity matrices based on raw patterns were significantly correlated with the theoretical bias structure (the largest Bonferroni-corrected p-value of Pearson correlation is 0.0007) and 51 ± 18% of the variance in the off-diagonal elements can be explained by the theoretical bias.

Second, averaging raw data 6 sec after events of interest over-estimates the similarity between neural patterns of adjacent events, an effect independent of the fMRI noise property. This is because the true HRF in the brain has a protracted time course regardless of how one analyzes the data. Thus the estimated patterns (we denote by ${\hat{\beta }}_{6}$) in this approach are themselves biased due to the mismatch between the implicit HRF that this averaging assumes and the real HRF. The expectation of ${\hat{\beta }}_{6}$ becomes $E\left[{\hat{\beta }}_{6}\right]=E\left[{\left(X_{6}^{T}X_{6}\right)}^{-1}X_{6}^{T}Y\right]=E\left[{\left(X_{6}^{T}X_{6}\right)}^{-1}X_{6}^{T}\left(X\beta +\epsilon \right)\right]={\left(X_{6}^{T}X_{6}\right)}^{-1}X_{6}^{T}X\beta$ instead of ***β***. Intuitively, **X** temporarily smears the BOLD patterns of neural responses close in time but ${\left(X_{6}^{T}X_{6}\right)}^{-1}X_{6}^{T}$ only averages the smeared BOLD patterns without disentangling the smearing. ${\hat{\beta }}_{6}$ thus mixes the BOLD activity patterns elicited by all neural events within a time window of approximately 12 sec (the duration of HRF) around the event of interest, causing over-estimation of the similarity between neural patterns of adjacent events. If the order of task conditions is not fully counterbalanced, this method would therefore still introduce into the estimated similarity matrix a bias caused by the structure of the task.

Similar effect can also be introduced if $\hat{\beta }$ is estimated with regularized least square regression [29]. Regression with regularization of the amplitude of $\hat{\beta }$ trades off bias in the estimates for variance (noise). On the surface, reducing noise in the pattern estimates may reduce the bias introduced into the similarity matrix. However, the bias in $\hat{\beta }$ itself alters the similarity matrix again. For example, in ridge regression, an additional penalization term **λ*β***^***T***^ ***β*** is imposed for ***β*** of each voxel. This turns estimates $\hat{\beta }$ to $\hat{\beta }={\left(X^{T}X+\lambda I\right)}^{-1}X^{T}Y$. The component contributed to $\hat{\beta }$ by the true signal ***Xβ*** becomes (**X**^***T***^ **X** + **λ*I***)^−**1**^ **X**^***T***^**X*β***. As **λ** increases, this component increasingly attributes neural activity triggered by other task events near the time of an event of interest to this event’s activity. Therefore, this method too would overestimate pattern similarity between adjacent events.

In all the derivations above, we have assumed for simplicity of illustration that the noise in all voxels has the same temporal covariance structure. In reality, the autocorrelation can vary over a large range across voxels (Fig 1G). So the structured noise in each voxel would follow a different distribution. Furthermore, the spatial correlation in noise means the noise in $\hat{\beta }$ is also correlated across voxels. Because noise correlation between voxels violates the assumption of Pearson correlation that observations (activity profiles of different voxels) are independent, the p-values associated with the correlation coefficients will not be interpretable. Although we made these simplified assumption for ease of illustration, in the model development below, variation of auto-correlation across voxels and spatial noise correlation are both considered in our proposed method.

### Bayesian RSA significantly reduces bias in the estimated similarity

As shown above, the covariance structure of the noise in the point estimates of neural activity patterns $\hat{\beta }$ leads to bias in the subsequent similarity measures. The bias can distort off-diagonal elements of the resulting similarity matrix unequally if the order of task conditions is not fully counterbalanced. In order to reduce this bias, we propose a new strategy that aims to infer directly the covariance structure ***U*** that underlies the similarity of neural patterns, using raw fMRI data. Our method avoids estimating $\hat{\beta }$ altogether, and instead marginalizes over the unknown activity patterns ***β*** without discarding uncertainty about them. The marginalization avoids the structured noise introduced by the point estimates, which was the central cause of the bias. Given that the bias comes not only from the experimental design but also from the spatial and temporal correlation in noise, we explicitly model these properties in the data. We name this approach Bayesian RSA (BRSA) as it is an empirical Bayesian method [30] for estimating ***U*** as a parameter of the prior distribution of ***β*** directly from data.

#### Direct estimation of similarity matrix while marginalizing unknown neural patterns

BRSA assumes a hierarchical generative model of fMRI data. In this generative model, the covariance structure ***U*** serves as a hyper-parameter that governs the distribution of ***β*** in an ROI, which in turn generates the observed fMRI signal **Y**. ***U*** can be different across ROIs, yielding different similarity structures among ROIs. Each voxel ***k*** has its own noise parameters, including auto-correlation coefficient ***ρ***_***k***_ and standard deviation ***σ***_***k***_ of the “shock” (the noise component at each time step that is unpredictable from the previous time step, sometimes termed “innovation”) in the first-order autoregressive (AR(1)) process used to model the auto-correlated noise in each voxel, and pseudo-SNR ***s***_***k***_ (we use the term ‘pseudo-SNR’ because the actual SNR depends on both the value of the shared covariance structure ***U*** and the voxel-specific scaling factor ***s***_***k***_. Therefore, ***s***_***k***_ are restricted to a fixed range while the magnitude of ***U*** is allowed to vary freely). Given these, (***σ***_***k***_***s***_***k***_)^**2**^***U*** is the covariance matrix of the distribution of the activity profile ***β***_⋅***k***_ in voxel ***k*** ($\beta_{\cdot k}\in R^{n_{C}\times 1}$). The model allows different signal and noise parameters across voxels to accommodate situations in which only a fraction of voxels in an ROI might have high response to tasks [29] and because the noise property can vary widely across voxels (e.g., Fig 1G). We denote the voxel-specific parameters ($\sigma_{k}^{2}$, ***ρ***_***k***_ and ***s***_***k***_) of all voxels together as ***θ***.

If the fMRI noise can be assumed to be independent across voxels [21], then for any single voxel ***k***, we can marginalize over the unknown latent variable ***β***_⋅***k***_ to obtain an analytic form of the likelihood of observing the fMRI data ***Y***_***k***_ in that voxel ***p***(***Y***_***k***_|**X**, ***U***, ***θ***_***k***_). Multiplying the likelihoods for all voxels will result in the likelihood for the entire dataset: ***p***(**Y**|**X**, ***U***, ***θ***). Note that this computation marginalizes over ***β***, avoiding altogether the secondary analysis on the point estimates $\hat{\beta }$ that is at the heart of traditional RSA. Through the marginalization, all the uncertainty about ***β*** is correctly incorporated into the likelihood. By searching for the optimal $\hat{U}$ and other parameters $\hat{\theta }$ that maximize the data likelihood, we can therefore obtain a much less biased estimate of ***U*** for the case of spatially independent noise [21].

However, as illustrated by [17], intrinsic fluctuation shared across brain areas that is not driven by stimuli can dominate the fMRI time series and influence the RSA result. If one labels any fluctuation not captured by the design matrix as noise, then intrinsic fluctuation shared across voxels can manifest as spatial correlation in the noise, which violates our assumption above. To reduce the impact of intrinsic fluctuation on the similarity estimation, we therefore incorporate this activity explicitly into the BRSA method, with inspiration from the GLM denoising approach [31, 32].

We start by assuming that the shared intrinsic fluctuation across voxels can be explained by a finite set of time courses, which we denote as ***X***_**0**_, and the rest of the noise in each voxel is spatially independent. If ***X***_**0**_ were known, the modulation ***β***_**0**_ of the fMRI signal **Y** by ***X***_**0**_ can be marginalized together with the response amplitude ***β*** to the experimental design matrix ***X*** (note that we still infer ***U***, the covariance structure of ***β***, not of ***β***_**0**_). Since ***X***_**0**_ is unknown, BRSA uses an iterative fitting procedure that alternates between a step of fitting the covariance structure ***U*** while marginalizing ***β***_**0**_ and ***β***, and a step of estimating the intrinsic fluctuation ***X***_**0**_ from the residual noise with principal component analysis (PCA). Details of this procedure are described in *Part 2 Model fitting procedure* of S1 Material.

**Fig 2:**
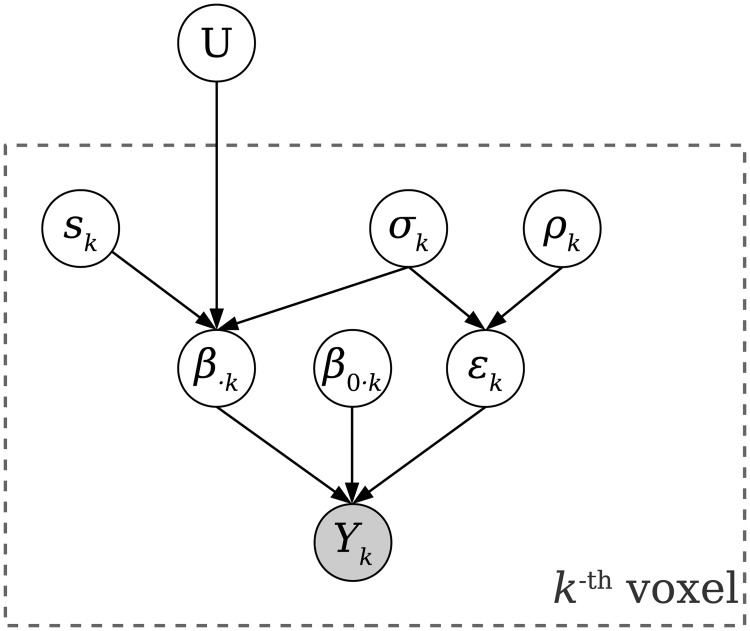
Generative model of Bayesian RSA. The covariance structure ***U*** shared across all voxels in an ROI is treated as a hyper-parameter of the unknown response amplitude ***β***. For voxel ***k***, the BOLD time series ***Y***_***k***_ are the only observable data. We assume ***Y***_***k***_ is generated by task-related activity amplitudes ***β***_⋅***k***_ (the ***k***-th column of ***β***), intrinsic fluctuation amplitudes ***β***_**0**⋅***k***_ and spatially independent noise ***ϵ***_***k***_: ***Y***_***k***_ = **X*β***_***k***_ + **X**_**0**_ ***β***_**0**⋅***k***_ + ***ϵ***_***k***_, where **X** is the design matrix and **X**_**0**_ is the set of time courses of intrinsic fluctuations. ***ϵ***_***k***_ is modeled as an AR(1) process with autocorrelation coefficient ***ρ***_***k***_ and noise standard deviation ***σ***_***k***_. ***β***_⋅***k***_ depends on the voxel’s pseudo-SNR ***s***_***k***_ and noise level ***σ***_***k***_ in addition to ***U***: ***β***_⋅***k***_ ∼ ***N***(**0**, (***s***_***k***_ ***σ***_***k***_)^**2**^ ***U***). By marginalizing over ***β***_⋅***k***_, ***β***_**0**⋅***k***_, ***σ***_***k***_, ***ρ***_***k***_ and ***s***_***k***_ for each voxel, we can obtain the likelihood function ***p***(***Y***_***k***_|**X**, **X**_**0**_, ***U***) and search for ***U*** which maximizes the total log likelihood $\text{log}\;p(Y|X,X_{0},U)=\sum_{k}^{n_{V}}\text{log}\;p(Y_{k}|X,X_{0},U)$ of the observed data **Y** for all ***n***_***V***_ voxels. The optimal $\hat{U}$ can be converted to a correlation matrix, representing the estimated similarity between patterns.

Since our goal is to estimate ***U***, voxel-specific parameters ***θ*** can also be analytically or numerically marginalized so that we only need to fit ***U*** for the marginal likelihood ***p***(**Y**|**X**, **X**_**0**_, ***U***). This reduces the number of free parameters in the model and further allows for the extension of estimating a shared representational structure across a group of participants, as shown later. Fig 2 shows a diagram of the generative model. More details regarding the generative model and the marginalization can be found in the Materials and Methods, under *Generative model of Bayesian RSA*.

The covariance matrix ***U*** can be parameterized by its Cholesky factor ***L***, a lower-triangular matrix. To find the $\hat{U}$ that best explains the data ***Y***, we first calculate the $\hat{L}$ that best explains the data by maximizing the marginal log likelihood:

$$\begin{array}{cc} \hat{L}= & \underset{L}{\text{arg}\;\text{max}}\;\text{log}\;p\left(Y|X,X_{0},L\right)\\ = & \underset{L}{\text{arg}\;\text{max}}\sum_{k}^{n_{V}}\text{log}\int \int \int d\beta_{\cdot k}d\beta_{0\cdot k}d\theta_{k}p\left(Y_{k}|X,X_{0},\beta_{\cdot k},\beta_{0\cdot k},\theta_{k}\right)\\ & \cdot p\left(\beta_{\cdot k}|L,\theta_{k}\right)p\left(\beta_{0\cdot k}\right)p\left(\theta_{k}\right) \end{array}$$ (6)

And then obtain the estimated covariance matrix

$$\begin{array}{c} \hat{U}=\hat{L}{\hat{L}}^{T} \end{array}$$ (7)

Once $\hat{U}$ is estimated (after the iterative fitting procedure for ***L*** and ***X***_**0**_), $\hat{U}$ is converted to a correlation matrix to yield BRSA’s estimation of the similarity structure in the ROI.

#### BRSA recovers simulated similarity structure

**Fig 3:**
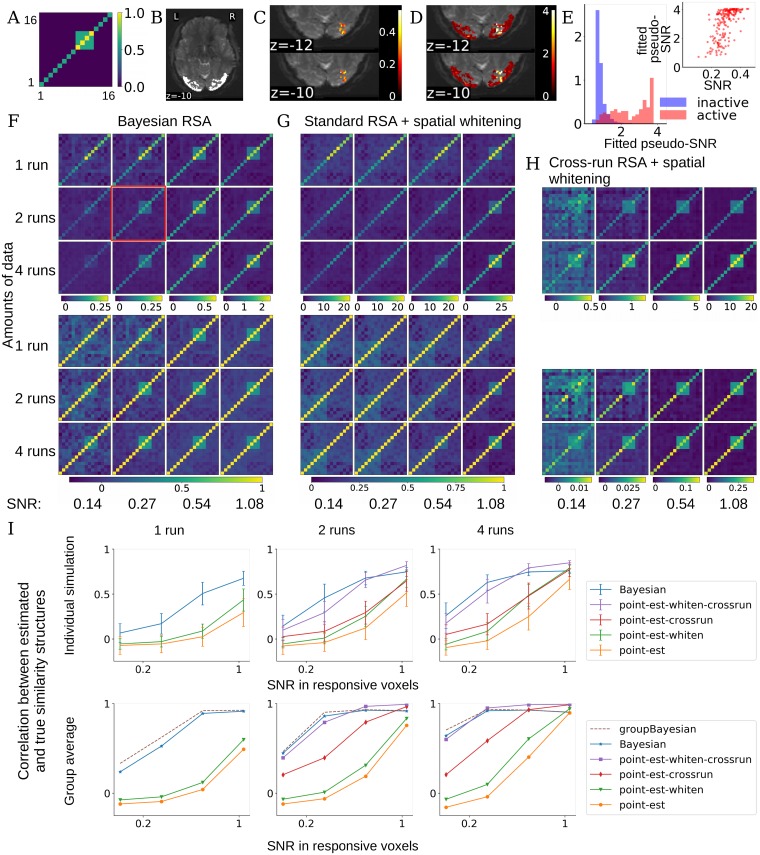
Performance of BRSA and other methods on simulated data. (**A**) We simulate task-related activation magnitude according to a multivariate normal distribution with a hypothetical “true” covariance structure *U* as displayed. (**B**) We use lateral occipital cortex (bright region) as an example ROI and resting state fMRI data from the Human Connectome Project as noise. (**C**) We multiplied the design matrix of the task in Fig 1A with the activity pattern simulated according to A and then added this “signal” to voxels in a cubical region of the ROI. The colors show the actual SNR of the added signal for one example simulated brain, corresponding to the plot circumvented by a red square in F. SNR here is defined as the ratio of standard deviation of the simulated signal to that of the noise (the time series of the resting state fMRI data): $\frac{\text{std}(X\beta_{\cdot k})}{\text{std}({\text{noise}}_{k})}$, where noise_***k***_ is the time series of the resting state fMRI data in voxel ***k***, treated as task-irrelevant noise in our simulation. (**D**) The pseudo-SNR map estimated by BRSA for the data with a true SNR map shown in C. The scale does not match the scale of true SNR, but the spatial pattern of SNR is recovered. The result corresponds to the simulation condition with red box in F. (**E**) The distributions of the fitted pseudo-SNR of task-active (pink) and inactive (blue) voxels are highly separable. The inset shows the SNR in active voxels and their fitted pseudo-SNR are significantly correlated. r = 0.62, p<1.4e-20. (**F**) Average covariance matrix (top) and similarity matrix (bottom) estimated by BRSA in the cubic area in C, across different SNR levels (columns) and different numbers of runs (rows). The average SNRs within the voxels with signals added (i.e., voxels with color in C) are displayed at the bottom. Note that we do not expect the values in the covariance or correlation matrix to scale linearly with SNR. The major effect of SNR is that the similarity structure becomes noisier as SNR decreases. (**G**) The corresponding result obtained by standard RSA based on activity patterns estimated within runs, which are spatially whitened. The major effect of SNR is that bias structure is stronger in the result as SNR decreases. (**H**) The corresponding result of RSA based on cross-correlating patterns estimated from separate runs, which are spatially whitened based on the residuals of all scanning runs. The major effect of SNR is noisier result and smaller correlational coefficients overall as SNR decreases. (**I**) Top: average correlation (mean ± std) between the off-diagonal elements of the estimated and true similarity matrices, for each method, across SNR levels (x-axis) and amounts of data (separate plots). Bottom: The correlation between the average estimated similarity matrix of each method (for GBRSA, this is the single similarity matrix estimated) and the true similarity matrix. “point-est”: methods based on point estimates of activity patterns; “-crossrun”: similarity based on cross-correlation between runs; “-whiten”: patterns were spatially whitened.

To test the performance of BRSA in a case where the ground-truth covariance structure is known, we embedded a structure into resting state fMRI data. Signals were simulated by first sampling response amplitudes according to a hypothetical covariance structure for the “16-state” task conditions (Fig 3A) for voxels assumed to respond to the task, and then weighting the design matrix of the task in Fig 1A by the simulated response amplitudes. The resulting simulated signals were then added to resting state fMRI data. In this way, the “noise” in the test data reflected the spatial and temporal structure of realistic fMRI noise. To make the estimation task even more challenging, we simulated a situation in which within the ROI (Fig 3B; we took the lateral occipital cortex as an ROI in this simulation, as an example) only a small proportion (∼ 4%) of voxels respond to the task conditions (Fig 3C). This is to reflect the fact that SNR often varies across voxels and that an ROI is often pre-selected based on anatomical criteria or independent functional localizer, which do not guarantee that all the selected voxels will have task-related activity. We evaluated the performance of BRSA at different levels of SNR and on different amounts of data (1, 2 and 4 runs of data, where each run includes fMRI data of 182±12 time points. The simulated ROI includes more than 4000 voxels.) The results of additional simulation in which signals were added in all voxels at lower SNR are similar, and provided in *Part 4 Performance of all methods when all voxels have task-related signals* of S1 Material.

In addition to comparing the performance of BRSA with traditional RSA, we also evaluated the performance of a few other approaches that have been proposed to improve the performance of RSA. One such approach is cross-run RSA [17–19]. Cross-run RSA calculates the similarity between patterns estimated from separate scanning runs, in contrast to the traditional RSA (here denoted as within-run RSA) which uses patterns estimated from the same scanning runs:

$$\begin{array}{c} C{}_{i,j_{\left(cross-run\right)}}\\ =\frac{1}{n_{runs}\left(n_{runs}-1\right)}\sum_{m=1}^{n_{runs}}\sum_{n=1,n\neq m}^{n_{runs}}\frac{\frac{1}{n_{V}}\sum_{k=1}^{n_{V}}\left({\hat{\beta }}_{ik}^{\left(m\right)}-\bar{{\hat{\beta }}_{i}^{\left(m\right)}}\right)\left({\hat{\beta }}_{jk}^{\left(n\right)}-\bar{{\hat{\beta }}_{j}^{\left(n\right)}}\right)}{\sigma_{{\hat{\beta }}_{i}^{\left(m\right)}}\sigma_{{\hat{\beta }}_{j}^{\left(n\right)}}} \end{array}$$ (8)

Where ***n***_***runs***_ is the total number of scanning runs; ${\hat{\beta }}_{ik}^{\left(m\right)}$ is the estimated activity of condition ***i*** at voxel ***k*** in the ***m***-th run, and $\bar{{\hat{\beta }}_{i}^{\left(m\right)}}$ and $\sigma_{{\hat{\beta }}_{i}^{\left(m\right)}}$ are the mean and standard deviation of the estimated pattern of condition ***i*** in the ***m***-th run calculated across voxels. The idea is that because the noises in the estimated patterns of different runs (e.g., ${\hat{\beta }}^{\left(m\right)}$ and ${\hat{\beta }}^{\left(n\right)}$) are independent, the expectation of the numerator of Eq 8 does not contain the bias term (**X**^***T***^ **X**)^−**1**^ **X**^***T***^**Σ**_***ϵ***_**X**(**X**^***T***^ **X**)^−**1**^ of Eq 5. However, the standard deviations in the denominator are still inflated by the noise in the estimates of patterns. Therefore this metric overall underestimates the pattern similarity [18]. This is reflected in the low correlation coefficients between any two conditions at low SNR. The resulting similarity matrix is also generally not positive semi-definite. For example, one may observe a result in which the pattern of condition ***i*** is more similar to that of condition ***j*** than to itself in cross-run RSA, or even negative correlation between patterns of the same condition across runs. Another approach is to spatially whiten the estimated patterns before calculating frequentist-based similarity metrics, either within-run or cross-run [19]. This serves to reduce the correlation between estimated activity profiles across voxels, making the whitened samples more conforming to the assumption of sample independence made by Pearson correlation. Following the procedure in [19], after estimating activity patterns $\hat{\beta }$ from each run, residuals of all runs were taken together to estimate a spatial covariance matrix of noise. Since there are more voxels than time points, the spatial noise covariance matrix is shrunk towards a diagonal matrix using the optimal shrinkage method proposed by Ledoit and Wolf [33], to make the covariance matrix invertible.

Fig 3F shows the average covariance structure and similarity matrix estimated by BRSA across 24 simulated subjects. The four-condition block in the simulated structure of Fig 3A can still be observed at low SNR and small amount of data. Spatial whitening improved the performance of both standard within-run RSA and cross-run RSA. Therefore, in Fig 3G and 3H we display the average covariance structure and similarity matrix estimated by within-run and cross-run RSA with spatial whitening. Even with spatial whitening, standard within-run RSA is still overwhelmed by the bias structure (Eq 5) in most of the simulations. Among the three displayed methods, cross-run RSA with spatial whitening has the least bias structure at the highest SNR. But at low SNR, as expected, noise impacts the result so much that the estimated similarity between patterns among condition 9-12 (refer to Fig 3A) can appear higher than the similarity between patterns of the same condition across runs (**3H**), making the result difficult to interpret. Furthermore, cross-run RSA can only be applied when there are more than one run of data. BRSA, in contrast, recovers the simulated similarity structure even at low SNR and small amount of data, and can be applied when there is only one run of data.

Fig 3I summarizes the average correlation between the off-diagonal elements of the estimated similarity matrix and those of the simulated similarity matrix. A 3-factor (RSA method, SNR and amounts of data) repeated-measure ANOVA on the simulation results with 2 and 4 runs of data shows significant main effect of RSA methods (F = 239.8, p<1e-47), main effect of SNR (F = 1647.6, p<4e-64), main effect of amounts of data (F = 76.1, p<1e-8), interaction between methods with SNR (F = 81.8, p<2e-83), interaction between amounts of data and SNR (F = 19.3, p<3e-9) and interaction among the three factors (F = 35.5, p<4e-49). Post-hoc paired t-tests between BRSA and every other method show that BRSA performs significantly better (highest p = 0.03). The second best method, cross-run RSA with spatial whitening, is also significantly better than the three other approaches (highest p = 2e-12). To confirm the impression that BRSA performs better at low SNR while cross-run RSA with spatial whitening performs better at high SNR, we performed an additional repeated-measures ANOVA with only these two approaches. The results indeed showed a significant interaction between RSA method and SNR (F = 3.0, p<4e-21). At the lowest SNR, the correlation between the simulated similarity structure and the similarity matrix estimated by within-run RSA with whitening is -0.057±0.056. This is consistent with the correlation between the simulated similarity structure and the theoretical spurious similarity structure of within-run RSA when assuming the analyzed data is pure white noise (-0.055±0.036), and further confirms our theoretical analysis.

The peak height of task-triggered response is often in the range of 0.1-0.5% of fMRI signal magnitudes in cognitive studies [34] while the noise level is often a few percents. This means that except when studying primary sensory stimulation, the SNRs expected in real studies are likely in the lower range in our simulation, where BRSA shows the most benefit.

#### Added bonus: Inferring pseudo-SNR map

Although the voxel-specific parameters ***θ*** are marginalized during fitting of the model, we can obtain their posterior distribution and estimate their posterior means. The estimated pseudo-SNR $\hat{s}$ is of particular interest, as it informs us of where the estimated representational structure is more strongly supported in the ROI chosen by the researcher. As shown in Fig 3D, the estimated pseudo-SNR map highly resembles the actual map of SNR in our simulated data in Fig 3C and they are significantly correlated. The correlation between estimated pseudo-SNR and actual SNR is 0.82 (p = 0) over all voxels in the ROI, and 0.62 (p<1.4e-20) in the active voxels with task-related signals added. The distributions of the estimated pseudo-SNR of task-active and inactive voxels are highly separable (Fig 3E).

#### Estimating shared representational similarity across participants

As mentioned above, BRSA can be extended to jointly fit the data of a group of participants, thus identifying the shared representational similarity structure that best explains the data of all participants. This is achieved by searching for a single ***U*** that maximizes the joint probability of observing all participants’ data (Group Bayesian RSA; GBRSA). The rationale of GBRSA is that it searches for the representational structure that best explains all the data. Using all the data to constrain the estimation of ***U*** reduces the variance of estimation for individual participants, an inspiration from hyper-alignment [22] and shared response model [23]. Fig 3I shows that the similarity structure recovered by GBRSA has slightly higher correlation with the true similarity structure than the average similarity structure estimated by other methods, across most of the SNR levels and amounts of data. Cross-run RSA with spatial whitening performs better only at the highest simulated SNRs. However, low average SNR is common in many brain areas and this is where (G)BRSA offers more power for detecting the true but weak similarity structure.

### Controlling for over-fitting: Model selection by cross-validation on left-out data

Although Fig 3 shows that BRSA reduces bias, it does not eliminate it completely. This may be due to over-fitting to noise. Because it is unlikely that the time course of intrinsic fluctuation ***X***_**0**_ and the design matrix ***X*** are perfectly orthogonal, part of the intrinsic fluctuation cannot be distinguished from task-related activity. Therefore, the structure of ***β***_**0**_, the modulation of intrinsic fluctuation, could also influence the estimated $\hat{U}$ when SNR is low.

**Fig 4:**
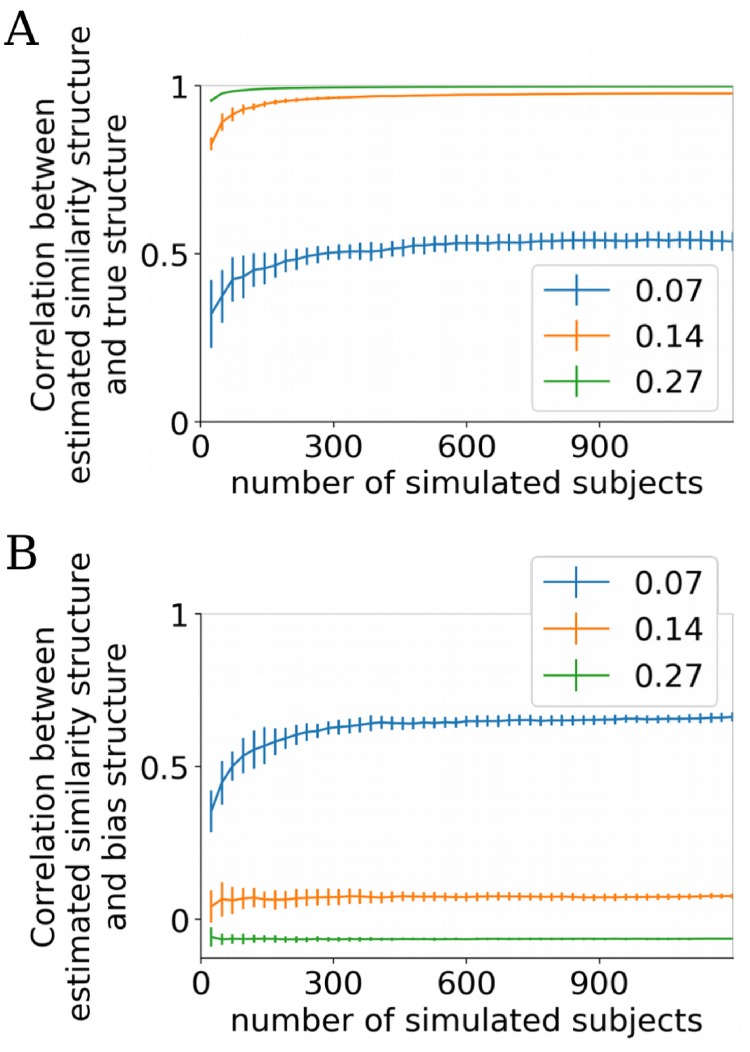
Limited performance of BRSA at very low SNR and small amount of data. (**A**) The average correlation between the off-diagonal elements of the estimated and the true similarity matrices (mean ± std) as the number of simulated subjects increases. Each simulated subject had one run of data. Legend shows average SNR in task-responsive voxels. Half of the voxels do not include any signal related to the design matrix. The correlation reaches asymptotic levels slightly below 1 with increasing numbers of participants except when the SNR is extremely low (0.07), indicating that the bias is not fully eliminated. (**B**) The average correlation between the estimated similarity matrix and the expected bias structure assuming white noise. The estimated similarity structure is most dominated by the bias structure at the lowest SNR simulated (0.07). The negative correlation at the highest SNR reflects the weak negative correlation between the true similarity structure and expected bias structure (-0.055).

For instance, in Fig 3F, at the lowest SNR and least amount of data (top left subplot), the true similarity structure is almost undetectable using BRSA. Is this due to large variance in the estimates, or is it because BRSA is still biased, but to a lesser degree than standard RSA? If the result is still biased, then averaging results across subjects will not remove the bias, and the deviation of the average estimated similarity structure from the true similarity structure should not approach 0. To test this, we simulated many more subjects by preserving the spatial patterns of intrinsic fluctuation and the auto-regressive properties of the voxel-specific noise in the data used in Fig 3, and generating intrinsic fluctuations that maintain the amplitudes of power spectrum in the frequency domain. To expose the limit of the performance of BRSA, we focused on the lower range of SNR and simulated only one run of data per “subject”. Fig 4A shows the quality of the average estimated similarity matrix with increasing number of simulated subjects. The average similarity matrices estimated by BRSA do not approach the true similarity matrix indefinitely as the number of subjects increase. Instead, their correlation saturates to a value smaller than 1. This indicates that the result of BRSA is still weakly biased, with the bias depending on the SNR. It is possible that as the SNR approaches 0, the estimated $\hat{U}$ is gradually dominated by the impact of the part of ***X***_**0**_ not orthogonal to ***X***. This bias is not due to underestimating the number of regressors in ***X***_**0**_ (see *Part 6 The effect of the number of nuisance regressors on BRSA performance* of S1 Material). We leave investigation of the source of this bias to future work. Empirically, the algorithm [35] we use to estimate the number of regressors in ***X***_**0**_ yields more stable and reasonable estimation than other methods we have tested (e.g., [36]). It should be noted that BRSA still performs much better than standard RSA, for which the correlation between the estimated similarity matrix and the true similarity matrix never passed 0.1 in these simulations.

The expected bias structure when spatial noise correlation exists is difficult to derive. We used (**X**^***T***^ **X**)^−**1**^ as a proxy to evaluate the residual bias in the estimated similarity using BRSA. As expected, when the SNR approached zero, the model over-fit to the noise and the bias structure increasingly dominated the estimated structure despite increasing the number of simulated participants (Fig 4B). This observation calls for an evaluation procedure to detect over-fitting in applications to real data, when the ground truth of the similarity structure is unknown.

One approach to assess whether a BRSA model has over-fit the noise is cross-validation. In addition to estimating ***U***, the model can also estimate the posterior mean of all other parameters, including the neural patterns ***β*** of task-related activity, ***β***_**0**_ of intrinsic fluctuation, noise variances ***σ***^**2**^ and auto-correlation coefficients ***ρ***. For a left-out testing data set, the design matrix ***X***_test_ is known given the task design. Together with the parameters estimated from the training data as well as the estimated variance and auto-correlation properties of the intrinsic fluctuation in the training data, we can calculate the log predictive probability of observing the test data. The unknown intrinsic fluctuation in the test data can be marginalized by assuming their statistical property stays unchanged from training data to test data. The predictive probability can then be contrasted against the cross-validated predictive probability provided by a null model separately fitted to the training data. The null model would have all the same assumptions as the full BRSA model, except that it would not assume any task-related activity captured by ***X***. When BRSA over-fits the data, the estimated spatial pattern $\hat{\beta }$ would not reflect the true response pattern to the task and is unlikely to be modulated by the time course in ***X***_test_. Thus the full model would predict signals that do not occur in the test data, and yield a lower predictive probability than the null model. The result of the full BRSA model on training data can therefore be accepted if the log predictive probability by the full model is higher than that of the null model significantly more often than chance.

Over-fitting might also arise when the assumed design matrix ***X*** does not correctly reflect task-related activity. When there is a sufficient amount of data but the design matrix does not reflect the true activity, the estimated covariance matrix $\hat{U}$ in BRSA would approach zero, as would the posterior estimates of $\hat{\beta }$. In this case as well, the full model would perform worse than the null model, because the form of the predictive likelihood automatically penalize more complex models.

**Fig 5:**
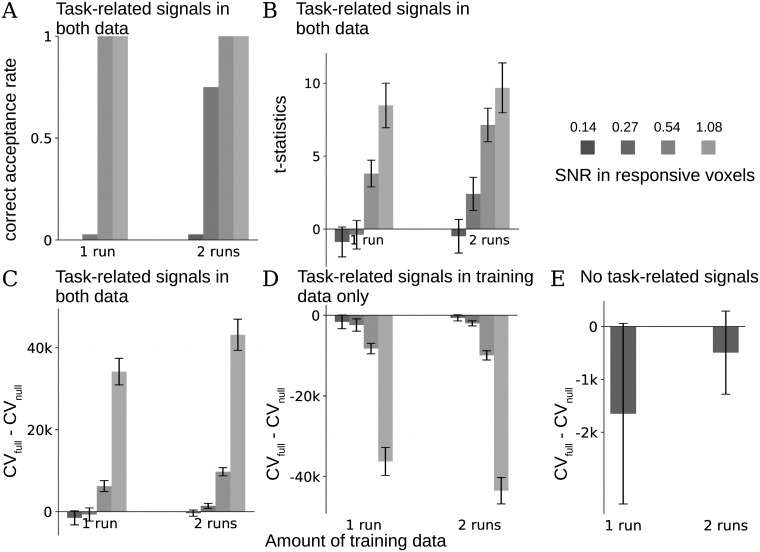
Cross-validation reduces the chance of false positive results. A group of 24 subjects with different SNRs were simulated as in Fig 3. 1 or 2 runs of data were used as training data and 1 left-out run was used as test data. The full BRSA model and a null model that assumes no task-related activity were fit to each simulated subject’s training data. Student’s t-test was performed on the differences between the cross-validation scores (the predictive log likelihoods for the test data) of the full model and null model across the simulated subjects to determine whether the full model should be accepted for each group of simulated subjects. This procedure was repeated on 36 different groups of simulated data (all using real fMRI data of non-overlapping subjects from HCP dataset as noise). (**A**) Task-related signals were added to both training and test data. The frequencies with which the full models were accepted based on the t-test (correct acceptance) are displayed for each simulation condition, grouped by the amounts of training data (1 or 2 runs). SNRs in the task-active voxels (about 4% of all) are displayed on the top-right and correspond to the SNRs in Fig 3. Full model was almost always rejected at the lowest simulated SNR. (**B**) Mean ± std of the t-statistics of the difference between cross-validation scores of the full and null models across simulated groups, for the corresponding amounts of data and SNR in A. (**C**) Mean ± std of the difference between the cross-validation scores of the full models and the null models across simulated groups in A. (**D**) Mean ± std of the difference between the cross-validation scores when only the training data but not test data were added with task-related signals. The statistical test correctly rejected the full model in all simulated groups. (**E**) Mean ± std of the difference between the cross-validation scores when neither training nor test data contain task-related signals. The statistical test correctly rejected the full model in all simulated groups.

We tested the effectiveness of relying on cross-validation to reject over-fitted results using the same simulation procedure as in Fig 3, and repeated this simulation 36 times, each time with newly simulated signals and data from a new group of participants in HCP [37] as “noise”. Each such simulated group represents one replication study. The ROI to extract “noise” from HCP dataset and the region to add signals were the same as in Fig 3. Fig 5A shows the rate of correct acceptance when both training and test data have signals. We counted each simulation in which the cross-validation score (log predictive probability) of the full BRSA model was significantly higher than the score of the null model (based on a one-sided student’s t-test at a threshold of ***α*** = 0.05) as one incidence of correct acceptance. When the SNR is high (at least 0.27 in the active voxels when there are two runs of training data, or at least 0.54 when there is one run of data), warranting reliable estimation of the similarity structures as indicated in Fig 3I, the cross-validation procedure selected the full model significantly more often than chance across simulation (the highest p = 0.03, binomial test with Bonferroni correction [38]). At low SNRs and with less training data (SNR below 0.27 when there is only one run of data or at 0.14 when there are two runs of data), the full model was almost never selected (p<9e-9), although in some cases the true similarity structure is still visible in the result of Fig 3F. This indicates that the cross-validation procedure is relatively conservative. The means and standard deviations of the t-statistics across simulated groups for all simulation configurations are displayed in Fig 5B. The differences in cross-validation scores between full and null models are displayed in Fig 5C. The reason that the null model can have a higher cross-validation score than the full model at low SNR is not only because of potential overfitting of the full model to the noise, but also because the full model’s log likelihood function has an additional log determinant term due to the inclusion of ***β***, which naturally penalizes for the extra complexity of the model compared to the null model (see Eqs (12), (13) and (15) in Materials and methods).

The cross-validation procedure also helps avoid false acceptance when activity patterns are not consistently reproducible across runs. To illustrate this, we simulated the case when signals are only added to the training data but not to the test data. Now, the full model was always rejected across the simulated SNR and amounts of data. Finally, when neither training data nor testing data included signal, the cross-validation procedure also correctly rejected the full model in all cases. Fig 5D and 5E illustrate the difference between cross-validation scores of full and null models for the two simulations, respectively. The advantage of the null model was smaller with 2 runs of training data in Fig 5E (t = -4.1, p = 2e-4, paired t-test), because in the full model the magnitudes of the posterior estimates of task-related activity patterns ${\hat{\beta }}_{\left(\text{post}\right)}$ are smaller with more training data, and this causes less mis-prediction for the test data.

### Extension: Decoding task-related activity from new data

**Fig 6:**
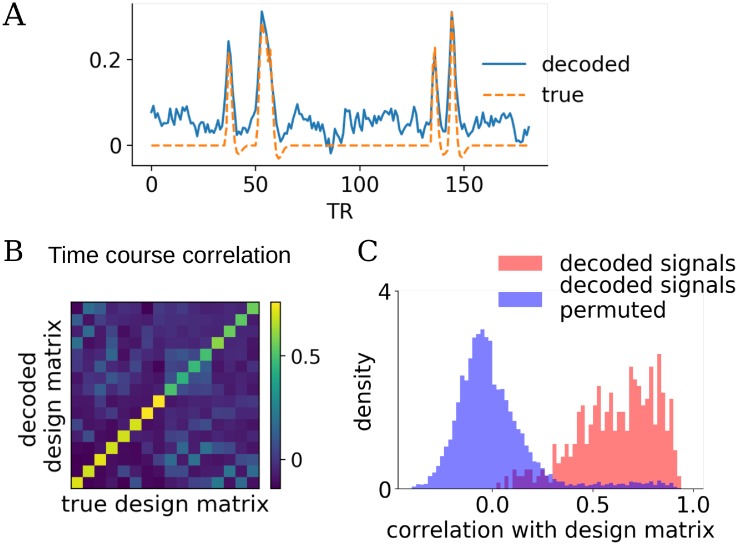
Decoding capabilities of the BRSA method. (**A**) Decoded task-related activity of the sixth condition from one simulated subject in one run of test data, and the true design matrix of that condition in the test data. The simulated data with the second highest SNR in Fig 3 were used. BRSA model was fitted to one run of training data. (**B**) Average correlation between the decoded signals for each task condition (rows) and the time courses for each condition in the design matrix used to simulate the test data (columns). (**C**) The distribution of correlation between the decoded signals of each condition with the time series in design matrix of the corresponding condition across 24 simulated subjects (pink), and the null distribution of correlation between condition-permuted decoded signals and design matrix (blue). The Bhattacharyya coefficient [39] between the two distributions is 0.36.

BRSA has a relatively rich model for the data: it attempts to model both the task-related signal and intrinsic fluctuation, and to capture voxel-specific SNR and noise properties. The covariance matrix ***U*** and the pseudo-SNR of each voxel serve as structured prior that make the estimation of $\hat{\beta }$ more precise. In addition to allowing cross-validation, the more precise estimation of activity patterns and the richer model of data also enable decoding of signals related to task conditions from new data. Similarly to the procedure of calculating cross-validated log likelihood, but without pre-assuming a design matrix for the test data, we can calculate the posterior mean of ${\hat{X}}_{\text{test}}$ and ${\hat{X}}_{0\text{test}}$ in the testing data. Fig 6A shows the decoded design matrix ${\hat{X}}_{\text{test}}$ for one task condition (condition 6 in Figs 1B and 6B) and one participant, using one run of training data with the second-highest SNR. Although our method decodes some spurious responses when there is no event of this task condition and that there is slight shift of baseline, overall the result captures most of the true responses in the design matrix. The average correlation between the decoded design matrix and the true design matrix is displayed in Fig 6B. High values on the diagonal elements indicate that overall, the decoder based on BRSA can recover the task-related signals well. These values are mostly beyond the range of the null distribution of correlations if task conditions were randomly shuffled (Fig 6C. The 7 percentile of the distribution of the values in the diagonal elements of Fig 6B is beyond the 93 percentile of the distribution of the shuffled correlations). The structure of the off-diagonal elements appears highly similar to those of the correlation structure between corresponding columns in the original design matrix(r = 0.82, p<6e-30) and also similar to the true pattern similarity structure we simulated (r = 0.30, p<1e-3). The resemblance to correlation in design matrix means that the signals corresponding to task conditions which often occur closely in time in training data are more likely to be confused when they are decoded from testing data. This is likely because the overlapping time courses between frequently co-occurring conditions make it difficult to distinguish which of two nearby events triggered the BOLD response during training, and reduced the accuracy of the posterior estimation of response patterns. We suspect that such confusion is not limited to decoding based on BRSA, but should be a general limitation of multi-variate pattern analysis of fMRI data: due to the slow smooth BOLD response, the more often the events of two task conditions occur closely in time in the training data, the more difficult it becomes for the classifier to discern their patterns. The resemblance to the true pattern similarity is not surprising since activity in conditions with similar neural patterns are expected to be more difficult to discern. The diagonal values were higher in conditions 1-8, because these conditions occurred for more times in the task design. This is expected, since more measurements lead to more reliable estimation of activity patterns.

## Discussion

In this paper, we demonstrated that bias can arise in the result of representational similarity analysis, a popular method in many recent fMRI studies. By analytically deriving the source of the bias with simplifying assumptions, we showed that it is determined by both the timing structure of the experiment design and the correlation structure of the noise (and task-unrelated neural activity) in the data. Traditional RSA is based on point estimates of neural activation patterns which unavoidably include high amounts of noise. The task design and noise property induce covariance structure in the noise of the pattern estimates. This structure, in turn, biases the covariance structure of these point estimates, and a bias persists in the similarity matrix. Such bias is especially severe when the SNR is low and when the order of the task conditions is not fully counterbalanced.

The bias demonstrated in this paper does not necessarily question the validity of all previous results generated by RSA. It does call for more caution when applying RSA to higher-level brain areas in which SNR of fMRI is typically low, and when the order of different task conditions cannot be fully counterbalanced. Counterbalancing a design could be done at different levels. The lowest level is randomizing the order of task conditions within a run. At higher levels, task sequences can also be randomized across runs and participants. If there are few measurements of each task condition within a run, randomizing trial order only within a run does not guarantee a perfectly counterbalanced design. The bias structure can then deviate from a diagonal matrix. If the same random sequence is used for all runs, or for all participants, small biases can persist across runs and participants and become a confound. Therefore, it is also important to use different task sequences across runs and participants when possible. Although such counterbalancing at different levels is desirable, it may not be achievable when studying high-level cognition, for instance in decision making tasks that involve learning or structured sequential decisions. These tasks often require (or impose) a specific relationship among conditions and events cannot be randomly shuffled.

To reduce this bias, especially for cases when counterbalancing task conditions is not possible, we proposed a Bayesian framework that interprets the representational structure as reflecting the shared covariance structure of activity levels across voxels. Our BRSA method estimates this covariance structure directly from data, bypassing the structured noise in the point estimates of activity profiles, and explicitly modeling the spatial and temporal structure of the noise. This is different from many other methods that attempt to correct the bias after it has been introduced. Interestingly, Lindquist et al. recently pointed out that when applying a sequence of processing steps to fMRI data, a later step can reintroduce nuisance effects that earlier steps attempted to remove. They therefore suggest combining all the processing steps into one single filtering step [40], which is similar in spirit to the method we propose here. Although the issues of performing multiple analyses steps sequentially arise in different context, these works together raise caution for developing new analyses on quantities estimated by existing processing pipelines. Furthermore, in the view of [40], regression analysis is one but many possible projections of the original data to a subspace. Therefore, other preprocessing steps can also potentially alter the covariance structure of noise in the point estimates of patterns, and consequently alter the bias structure in standard RSA.

In addition to inferring the representational similarity structure, our method also infers activation patterns (as an alternative to the traditional GLM), SNR for different voxels, and even the “design matrix” for data recorded without knowledge of the underlying conditions. The inferred activation patterns are regularized not only by the SNR, but also by the learned similarity structure. The inference of an unknown “design matrix” allows one to uncover uninstructed task conditions (e.g., in free thought) using the full Bayesian machinery and all available data.

In a realistic simulation using real fMRI data as background noise, we showed that BRSA generally outperforms standard RSA and cross-run RSA, especially when the SNR is low and when the amount of data is limited, making our method a good candidate in scenarios of low SNR (e.g., higher order cortices) and difficult-to-balance tasks (e.g., learning and sequential decision making). Because temporal and spatial correlations also exist in the noise of data from other neural recording modalities, the method can also be applied to other types of data when the bias in standard RSA is of concern. To detect overfitting to noise, the difference between the cross-validated score of the full model of BRSA and a null model can serve as the basis for model selection. We further extended the model to allow for estimating the shared representational structure across a group of participants. This shared structure can be very useful in model testing. As suggested by [41], the correlation between similarity matrices estimated from individual participants’ data and the group average similarity matrix reflects the “noise ceiling”: the expected correlation one can achieve between the similarity matrix from an unknown “true” computational model and the similarity matrix from data, given the amount of noise in the data. The shared similarity matrix obtained through the joint fitting of GBRSA can be used in place of the group average similarity matrix for the purpose of calculating this noise ceiling.

Prior proposals suggested the use of similarity between patterns estimated from separate scanning runs (cross-run RSA) [18, 19, 42], in order to overcome “pattern drift” [18], which can be seen as being caused by an interaction between the study design and auto-correlated fMRI noise. The inner product between noise pattern estimates from separate runs is indeed theoretically unbiased. Cross-run RSA was also proposed for assessing the extent to which brain patterns can discriminate between different conditions [43]. In our simulations, cross-run RSA generally performs better than within-run RSA, but worse than BRSA. However, after spatial whitening of the point estimates of activity patterns, cross-run RSA outperforms BRSA at high SNR, with the results of both methods very close to the true similarity structure in this case. On the other hand, at low SNR, BRSA performs better. The limitation of cross-run RSA is that even though the cross-run covariance matrix is unbiased, the magnitude of cross-run correlation is underestimated. Additionally, the high noise in the pattern estimates can also lead to results that are difficult to interpret, such as an anti-correlation between estimated patterns of the same condition from different runs. In general, it is not guaranteed that cross-run RSA will obtain a measure of distance that satisfies the triangle inequality. In contrast, although BRSA is not fully unbiased, it does guarantee that the estimated similarity matrix is positive semi-definite, and that an interpretable distance metric can be derived by subtracting the similarity matrix from 1. In sum, it is difficult to predict whether BRSA or cross-run RSA with spatial whitening will be more suitable for any specific study and brain area of interest since their performance depends on SNR and both methods have limitations. Nonetheless, based on our results, both approaches should always be favored over traditional within-run RSA.

While spatial whitening might be desirable for the purpose of cross-run RSA since it improves the performance, one should take caution when interpreting activity pattern after whitening. This is because spatial whitening remixes data across voxels and risks moving task-related signals from voxels responding to a task to non-responsive voxels. Instead of performing spatial whitening, BRSA estimates several time series ***X***_**0**_ that best explain the correlation of noise between voxels, and marginalizes over their modulations of activity in each voxel. Therefore, BRSA can capture spatial noise correlation without the risk of misattributing signals across voxels.

In our study, we did not directly compare BRSA to cross-validated Mahalanobis distance [19] because these two methods are fundamentally different measures: BRSA aims to estimate the correlation between patterns, which is close to the cosine angle between two patterns vectors [44–46]; in contrast, Mahalanobis distance aims to measure the distance between patterns. Nonetheless, given the theoretical soundness of the cross-validated Mahalanobis distance and the possibility of statistically testing the distances against 0, it could also be a good alternative to BRSA when there are multiple runs in a study.

Our BRSA method is closely related to pattern component modeling (PCM) [10, 47]. A major difference is that PCM models the point estimates $\hat{\beta }$ after a GLM analysis while BRSA models fMRI data **Y** directly. The original PCM [10] in fact considered the contribution of noise in pattern estimates to the similarity matrix, but assumed that the noise in $\hat{\beta }$ is i.i.d across task conditions. This means that the bias in the covariance matrix was assumed to be restricted to a diagonal matrix. We showed here that when the order of task conditions cannot be fully counterbalanced, such as in the example in Fig 1, this assumption is violated and the bias cannot be accounted for by methods such as PCM. Another difference is that BRSA explicitly models spatial noise correlation, which can improve the results (see Figure **3** of S1 Material).

If the covariance structure of the noise **Σ**_***ϵ***_ were known, the diagonal component of the noise covariance structure assumed in PCM [10] could be replaced by the bias term (**X**^***T***^ **X**)^−**1**^ **X**^***T***^**Σ**_***ϵ***_ **X**(**X**^***T***^ **X**)^−**1**^ to adapt PCM to estimate the covariance structure $\hat{U}$ that best explains $\hat{\beta }$ [47], ignoring spatial noise correlation. However, as shown in Fig 1G, different voxels can have a wide range of different autocorrelation coefficients, therefore assuming a single **Σ**_***ϵ***_ for all voxels may be over-simplifying. PCM also assumes all voxels within one ROI have equal SNR. However, typically only a small fraction of voxels exhibit high SNR [29]. Therefore, it is useful to model the noise property and SNR of each voxel individually. Relatedly, Alink et al. proposed to model the bias structure in the similarity matrix as a polynomial function of the temporal distance between each pair of conditions and regress out such structure [18]. This regression approach is plausible when each task condition contains a single event. If one were to accept the simplifying assumptions above, a more principled choice for regressing out the bias structure would use the analytic form of the bias structure we derived above as a regressor.

BRSA comes with the ability to select between a full model and null model based on cross-validated log likelihood, and can be applied to fMRI decoding. PCM can evaluate the likelihood of a few fixed candidate representational structures given by different computational models. It can also estimate the additive contributions of several candidate pattern covariance structures to the observed covariance structure. These options are not yet available in the current implementation of BRSA. Combining the strengths of PCM and BRSA is an interesting direction for future research. Another future direction is to cross-validate full and null models on the data of left-out subjects, instead of cross-validating within subjects on a left-out run of data.

Many aspects of flexibility may be incorporated to BRSA. For example, the success of the analysis hinges on the assumption that the hemodynamic response function (HRF) used in the design matrix correctly reflects the true hemodynamics in the region of interest, but the HRF in fact varies across people and across brain regions [48, 49]. Jointly fitting the shape of the HRF and the representational structure using BRSA may thus improve the estimation. In addition, it is possible that even if the landscape of activity patterns for a task condition stays the same, the global amplitude of the response pattern may vary across trials due to repetition suppression [50–52] and attention [53, 54]. Allowing global amplitude modulation of patterns associated with a task condition to vary across trials might capture such variability and increase the power of the method.

Finally, our simulations revealed that BRSA is not entirely unbiased, that is, the results cannot be improved indefinitely by adding more subjects. This bias is not a consequence of the number of components estimated by the algorithm we chose [35] (see *Part 6* of S1 Material), and further investigation is needed to understand the source of this remaining bias. The residual bias occurs when SNR is very low and may be due to overfitting of the model to noise. Fortunately, the cross-validation procedure we provided helps to detect overfitting when the SNR is too low. When this happens, it is advisable to focus on taking measures to improve the design of study. Ultimately, task designs that are not fully counterbalanced and low SNR in fMRI data are two critical factors that cause bias in traditional RSA and impact the power of detecting similarity structure in neural representations. Carefully designing tasks that balance the task conditions as much as possible, using different randomized task sequences across runs and across participants, and increasing the number of measurements, are our first line of recommendations. In the analysis phase of the project, one can then use BRSA.

## Materials and methods

### Ethics statement

The fMRI data used in the current manuscript came from two sources. Part of them came from a previously published study, which was approved by the Princeton Institutional Review Board and all subjects gave informed written consent prior to participation. The other part came from open access data of the Human Connectome Project (https://www.humanconnectome.org/).

### Generative model of Bayesian RSA

The detailed derivation of the generative model, the model fitting procedure, model selection based on cross-validation and decoding task-related signals are in the *Parts 1, 2 and 3* of S1 Material. Here we provide the major assumptions and the formula of the likelihood of fMRI data in our model.

Our generative model of fMRI data follows the general assumption of GLM. In addition, we model spatial noise correlation by a few time series ***X***_**0**_ shared across all voxels. The contribution of ***X***_**0**_ to the ***k***-th voxel is ***β***_**0**⋅***k***_. Thus, for voxel ***k***, we assume that

$$Y_{k}=X\beta_{\cdot k}+X_{0}\beta_{0\cdot k}+\epsilon_{k}$$ (9)

***Y***_***k***_ is the time series of voxel ***k***. ***X*** is the design matrix shared by all voxels. ***β***_⋅***k***_ is the response amplitudes of the voxel ***k*** to all the task conditions. ***ϵ***_***k***_ is the residual noise in voxel ***k*** which cannot be explained by either ***X*** or ***X***_**0**_. We assume that ***ϵ*** is spatially independent across voxels, and all the correlation in noise between voxels are captured by the shared intrinsic fluctuation ***X***_**0**_.

We use an AR(1) process to model ***ϵ***_***k***_: for the ***k***-th voxel, we denote the noise at time ***t*** > **0** as ***ϵ***_***t***,***k***_, and assume

$$\begin{array}{c} \epsilon_{t,k}=\rho_{k}\epsilon_{t-1,k}+\eta_{t,k},\;\eta_{t,k}∼N\left(0,\sigma_{k}^{2}\right) \end{array}$$ (10)

where $\sigma_{k}^{2}$ is the variance of the “shock” (innovation), the component at each time point ***t*** that is independent from ***ϵ***_***t***−**1**,***k***_, and ***ρ***_***k***_ is the autoregressive coefficient for the ***k***-th voxel.

We assume that the covariance of the multivariate Gaussian distribution from which the activity amplitudes ***β***_***k***_ are generated has a scaling factor that depends on its pseudo-SNR ***s***_***k***_:

$$\begin{array}{c} \beta_{\cdot k}∼N\left(0,{\left(s_{k}\sigma_{k}\right)}^{2}U\right). \end{array}$$ (11)

This is to reflect the fact that not all voxels in an ROI have equal SNR.

We further use Cholesky decomposition to parametrize the covariance structure ***U***: ***U*** = ***LL***^***T***^, where ***L*** is a lower triangular matrix.

With the above assumption of the distributions of activity profiles and noise in each voxel, after marginalizing ***β***_**0**⋅***k***_ and ***β***_⋅***k***_, we have

$$\begin{array}{c} p(Y_{k}|X,X_{0},L,\sigma_{k},\rho_{k},s_{k})\\ \;=\int \int p\left(Y_{k}|\beta_{\cdot k},\beta_{0\cdot k},X,X_{0},\sigma_{k},\rho_{k}\right)p\left(\beta_{0\cdot k}\right)p\left(\beta_{\cdot k}|s_{k},\sigma_{k},L\right)d\beta_{0\cdot k}d\beta_{\cdot k}\\ \;\propto {\left(2\pi \right)}^{-\frac{n_{T}-n_{0}}{2}}|\Sigma_{\epsilon_{k}}^{-1}{|}^{\frac{1}{2}}|X_{0}^{T}\Sigma_{\epsilon_{k}}^{-1}X_{0}{|}^{-\frac{1}{2}}{\left|\Lambda_{k}^{*}\right|}^{\frac{1}{2}}\\ \;\cdot \text{exp}[-\frac{1}{2}\left(\frac{1}{\sigma_{k}^{2}}Y_{k}^{T}A_{k}^{*}Y_{k}-\mu_{k}^{*T}\Lambda_{k}^{*-1}\mu_{k}^{*}\right)] \end{array}$$ (12)

In the equation above, $\Sigma_{\epsilon_{k}}^{-1}$ is the temporal autocorrelation matrix of the AR(1) noise in voxel *k*. $A_{k}^{*}=\sigma_{k}^{2}\left(\Sigma_{\epsilon_{k}}^{-1}-\Sigma_{\epsilon_{k}}^{-1}X_{0}{\left(X_{0}^{T}\Sigma_{\epsilon_{k}}^{-1}X_{0}\right)}^{-1}X_{0}^{T}\Sigma_{\epsilon_{k}}^{-1}\right)=A_{k}-A_{k}X_{0}{\left(X_{0}^{T}A_{k}X_{0}\right)}^{-1}X_{0}^{T}A_{k}$, where $A_{k}=A\left(\rho_{k}\right)=\sigma_{k}^{2}\Sigma_{\epsilon_{k}}^{-1}$. $\Lambda_{k}^{*}={\left(i+s_{k}^{2}L^{T}X^{T}A_{k}^{*}XL\right)}^{-1}$ and $\mu_{k}=\frac{s_{k}}{\sigma_{k}}\Lambda_{k}^{*}L^{T}X^{T}A_{k}^{*}Y_{k}$.

***σ***_***k***_ can be further analytically marginalized. ***s***_***k***_ and ***ρ***_***k***_ cannot be analytically marginalized. But we can numerically marginalize them by weighted sum of the likelihood at ***n***_***l***_ × ***n***_***m***_ discrete grids **{*ρ***_***kl***_, ***s***_***km***_**}** (**0** < ***l*** < ***n***_***l***_, **0** < ***m*** < ***n***_***m***_) with each grid representing one area of the parameter space of (***ρ***, ***s***). The weights ***w***(***ρ***_***kl***_, ***s***_***km***_) reflect the prior probabilities of the two parameters in the area represented by **{*ρ***_***kl***_, ***s***_***km***_**}**. We assume uniform prior of ***ρ*** in (-1, 1). All the simulations in this paper used an exponential distribution as prior for ***s***. Alternative forms of priors such as uniform in (0, 1), log normal distribution, and Delta distribution of fixed pseudo-SNR are also implemented in the tool. A comparison of the impact of different forms of prior assumption on BRSA’s performance is provided in *Part 5 Comparison of BRSA with different assumptions of the prior of pseudo-SNR* of S1 Material. This procedure yields the marginalized log likelihood for each voxel:

$$\begin{array}{c} p(Y_{k}|X,X_{0},L)\\ \;\approx \sum_{l=1}^{n_{l}}\sum_{m=1}^{n_{m}}p\left(Y_{k}|X,X_{0},L,\rho_{kl},s_{km}\right)w\left(\rho_{kl},s_{km}\right)\\ \;\propto \sum_{l=1}^{n_{l}}\sum_{m=1}^{n_{m}}{\left(2\pi \right)}^{-\frac{n_{T}-n_{0}}{2}}{\left(1-\rho_{kl}^{2}\right)}^{\frac{n_{r}}{2}}|X_{0}^{T}A_{kl}X_{0}{|}^{-\frac{1}{2}}{\left|\Lambda_{klm}^{*}\right|}^{\frac{1}{2}}\Gamma \left(\frac{n_{T}-n_{0}}{2}-1\right)\\ \;\cdot {\left[\frac{Y_{k}^{T}A_{kl}^{*}Y_{k}-s_{km}^{2}Y_{k}^{T}A_{kl}^{*}XL\Lambda_{klm}^{*}L^{T}X^{T}A_{kl}^{*}Y_{k}}{2}\right]}^{1-\frac{n_{T}-n_{0}}{2}}w\left(\rho_{kl},s_{km}\right) \end{array}$$ (13)

where $A_{kl}^{*}$ is $A_{k}^{*}$ assessed at parameter grid ***ρ***_***kl***_, $\Lambda_{klm}^{*}$ is $\Lambda_{k}^{*}$ assessed at grid **{*ρ***_***kl***_, ***s***_***km***_**}**.

Because we made the assumption that ***ϵ***_***k***_ is independent across voxels, the log likelihood for all data is the sum of the log likelihood for each voxel.

$$\begin{array}{c} \text{log}\;p\left(Y|X,X_{0},L\right)=\sum_{k=1}^{n_{V}}\text{log}\;p\left(Y_{k}|X,X_{0},L\right). \end{array}$$ (14)

For the null model, the likelihood for each voxel after marginalizing ***β***_**0 · *k***_ and $\sigma_{k}^{2}$ can be similarly derived,

$$\begin{array}{c} p(Y_{k}|X_{0},\rho_{k})\\ \propto {\left(2\pi \right)}^{-\frac{n_{T}-n_{0}}{2}}{\left(1-\rho_{k}^{2}\right)}^{\frac{n_{r}}{2}}{\left|X_{0}^{T}A_{k}X_{0}\right|}^{-\frac{1}{2}}\\ \cdot \Gamma \left(\frac{n_{T}-n_{0}}{2}-1\right){\left[\frac{Y_{k}^{T}A_{k}^{*}Y_{k}}{2}\right]}^{1-\frac{n_{T}-n_{0}}{2}} \end{array}$$ (15)

and the total log likelihood can be calculated similarly by numerically marginalizing ***ρ***_***k***_ and summing the log likelihood for all voxels.

### Data processing and analysis

Data used in Fig 1B are from the experiments of Schuck et al. [24], following the same preprocessing procedure as the original study. The fMRI data were acquired at TR = 2.4s. Data of 24 participants were used. Their design matrices were used for all the following analyses and simulations. Data in Figs 1E, 1G and 3 were preprocessed data obtained from Human Connectome Project (HCP) [37]. The first 24 participants who have completed all 3T protocols and whose data were acquired in quarter 8 of the HCP acquiring period without image quality issues were selected for analysis in Fig 3. Data from 864 participants without image quality issues in HCP were used in the analysis in Fig 5. Each participants in the HCP data have 2 runs of resting state data with left-right phase encoding direction and 2 runs with right-left phase encoding direction. Denoising pipeline ICA-AROMA [55] with default parameters was performed on each run of data. The time series of the aggressively denoised data were resampled at the same TR as the design matrix and truncated to the length of each run in that design matrix before further analysis (all results were similar without ICA-AROMA denoising). Each run contained 182 ± 12 time points.

$\hat{\beta }$ point estimates in Fig 1 were obtained with AFNI’s *3ddeconvolve* [56]. The design matrices were set up by convolving the stereotypical double-Gamma HRF in SPM [57] with event time courses composed with impulses lasting for the duration of the participants’ reaction time. AR(1) coefficients in Fig 1G were estimated after upsampling the fMRI time series in the HCP data to the TR in Schuck et al. [24] and linear detrending. Upsampling is to reflect the lower temporal resolution more typically employed in task-related fMRI studies.

In the experiments of Fig 3, lateral occipital cortex was chosen as the ROI, which included 4802 ± 31 (mean ± standard deviation) voxels. Task related signals were only added to voxels within a bounding box (Fig 3C) of which the coordinates satisfy 25 < ***x*** < 35, -95< ***y*** < −5 and -15< ***z*** <5. 181.9±0.3 voxels fell within this bounding box. To generate activity patterns at different SNR, ***β*** were sampled independently from a multivarite Gaussian distribution with the covariance matrix in Fig 3A for each voxel within the bounding box mentioned above (resting state data from HCP), and were scaled by one values in 1, 2, 4, 8 times the standard deviation of the detrended noise in that voxel. The design matrix ***X*** mentioned above were then multiplied with ***β*** for each voxel and added to the noise. Different random seeds were used in the simulation for different subjects, different SNR levels and different amounts of simulated data. But the same simulated data were used across different RSA analysis methods. Due to the small magnitude of the design matrix, the resulting ratios of the standard deviation of the simulated signals ***Xβ***_⋅***k***_ to that of the detrended noise were on average 0.14, 0.27, 0.54 and 1.08 within the bounding box (Fig 3C). To estimate the similarity matrix of this task involving 16 conditions, the fitting time on an Intel Xeon processor employing 12 CPUs is 751±105 s, 1274±414 s and 2206±564 s, for data of 1 run, 2 runs and 4 runs, respectively. This suggests that our method is practically feasible even for relatively large ROIs. To evaluate the performance of the recovered correlation structure by different methods, the off-diagonal elements of the similarity matrix recovered from data of each simulated participant was correlated with those elements of the ideal similarity matrix to yield the top panel of Fig 3I. The top panel reflects the correlation of individual results. The bottom panel reflects the correlation of average results over simulated participants.

In order to make fair comparison with BRSA which considers temporal auto-correlation in noise, all the point estimates of $\hat{\beta }$ by other methods in Fig 3 were performed with restricted maximum likelihood estimation, which model the auto-correlation in noise. AR(1) parameters of each voxel were estimated after initial regular regression. The AR(1) parameters were used to re-compute the temporal noise covariance matrices for each voxel and $\hat{\beta }$ were calculated again assuming these noise covariance matrices. To account for task-irrelevant time courses in the data, extra nuisance regressors were included in all methods based on point estimates of activity patterns. These included Legendre polynomial functions of volume index, up to the fourth order, to model slow drift of fMRI signals, and the first three principal components of the fMRI data in each of white matter and ventricles, to capture intrinsic fluctuations. The principal components were also included as nuisance regressors for BRSA. When spatial whitening of $\hat{\beta }$ was performed, residuals of fitting from all runs of data from the same simulated subject were concatenated in time. One covariance matrix **Σ**_***spatial***_ across voxels was then estimated from these residuals using the optimal shrinkage method [33] implemented in scikit-learn [58]. This covariance matrix was then used to whiten the estimated pattern of all the runs. That is, $\hat{\beta }\Sigma_{spatial}^{-\frac{1}{2}}$ of each run were treated as the spatially whitened pattern estimates. When performing within-run RSA, the estimated patterns of each run (being spatially whitened or not) were averaged over the simulated runs before subjecting to a Pearson correlation. When performing cross-run RSA, the correlations were calculated between the estimated patterns corresponding to any two conditions from two different runs. This calculation was repeated for each combination of two runs in the data. And finally all the correlation coefficients between the two conditions calculated over all pairs of runs were averaged (e.g., 12 cross-run similarity matrix would be calculated from 4 runs of data and averaged to generate one matrix).

To simulate the fMRI noise in Fig 4, we first estimated the number of principal components to describe the spatial noise correlation in the 24 resting state fMRI data from HCP database using the algoritm of Gavish and Donoho [35]. The spatial patterns of these principal components were kept fixed as the modulation magnitude ***β***_**0**_ by the intrinsic fluctuation. AR(1) parameters for each voxel’s spatially indepndent noise were estimated from the residuals after subtrating these principal components. For each simulated subject, time courses of intrinsic flucutations were newly simulated by scrambling the phase of the Fourier transformation of the ***X***_**0**_ estimated from the real data, thus preserving the amplitudes of their frequency spectrum. AR(1) noise were then added to each voxel with the same parameters as estimated from the real data. To speed up the simulation, only 200 random voxels from the ROI in Fig 3B were kept for each participant in these simulations. Among them, 100 random voxels were added with simulated task-related signals. Thus, each simulated participant has different spatial patterns of ***β***_**0**_ due to the random selection of voxels. 500 simulated datasets were generated based on the real data of each participant, for each of the three SNR levels. In total 36000 subjects were simulated. The simulated pool of subjects were sub-divided into bins with a fixed number of simulated subjects ranging from 24 to 1200. The mean and standard deviation of the correlation between the true similarity matrix and the average similarity matrix based on the subjects in each bin were calculated, and plotted in Fig 4A.

All SNRs in Figs 3 and 4 were calculated post hoc, using the standard deviation of the added signals in the bounding box region devided by the standard deviation of the noise in each voxel, and averaged across voxels and simulated subjects for each level of simulation.

## Supporting information

S1 Material Supplementary materials. Part 1: Details of the generative model of Bayesian RSA. Part 2: Model fitting procedure. Part 3: Model selection and decoding task-related signals. Part 4: Performance of all methods when all voxels have task-related signals. Part 5: Comparison of BRSA with different assumptions of the prior of pseudo-SNR. Part 6: The effect of the number of nuisance regressors in BRSA performance. Part 7: Cross-validation with less stringent criterion.
(PDF)
