# Supplementary material for: Representational structure or task structure? Bias in neural representational similarity analysis and a Bayesian method for reducing bias
Source: PLoS Comput Biol. 2019 May 24;15(5):e1006299. doi: 10.1371/journal.pcbi.1006299 (PMC6553797; doi:10.1371/journal.pcbi.1006299)
Supplement: S1 Material — Part 1: Details of the generative model of Bayesian RSA. Part 2: Model fitting procedure. Part 3: Model selection and decoding task-related signals. Part 4: Performance of all methods when all voxels have task-related signals. Part 5: Comparison of BRSA with different assumptions of the prior of pseudo-SNR. Part 6: The effect of the number of nuisance regressors in BRSA performance. Part 7: Cross-validation with less stringent criterion. (PDF) [file pcbi.1006299.s001.pdf]

S1 Supplementary Materials

Representational structure or task structure? Bias in neural representational similarity analysis and a Bayesian method for reducing bias

Ming Bo Cai[1*], Nicolas W. Schuck [3,4,1], Jonathan W. Pillow[1,2], Yael Niv[1,2],

1 Princeton Neuroscience Institute, Princeton University, Princeton, NJ 08544 USA

2 Department of Psychology, Princeton University, Princeton, NJ 08544 USA

3 Max Planck Research Group Neurocode, Max Planck Institute for Human Development, 14195 Berlin, Germany

4 Max Planck UCL Centre for Computational Psychiatry and Ageing Research, 14195 Berlin, Germany

* mcai@princeton.edu

Part 1 Details of the generative model of Bayesian RSA

Our generative model of fMRI data follows the general assumption of GLM. In addition, we model spatial noise correlation by a few time series X_0 shared across all voxels. The contribution of X_0 to the k-th voxel is $\beta_{0\cdot k}$. Thus, for voxel k, we assume that

$$Y_k = X\beta_{\cdot k} + X_0\beta_{0\cdot k} + \epsilon_k \tag{1}$$

Y_k is the time series of voxel k. X is the design matrix shared by all voxels. $\beta_{\cdot k}$ is the response amplitudes of the voxel k to all the task conditions. ϵ_k is the residual noise in voxel k which cannot be explained by either X or X_0. We assume that ϵ is spatially independent

across voxels, and all the correlation in noise between voxels are captured by the shared intrinsic fluctuation X_0.

We use an AR(1) process to model ϵ_k: for the k-th voxel, we denote the noise at time $t > 0$ as $\epsilon_{t,k}$, and assume

$$\epsilon_{t,k} = \rho_k \epsilon_{t-1,k} + \eta_{t,k}, \quad \eta_{t,k} \sim \mathbf{N}(0, \sigma_k^2) \tag{2}$$

where σ_k^2 is the variance of the "innovation" ("shock"), the component at each time point t that is independent from $\epsilon_{t-1,k}$, and ρ_k is the autoregressive coefficient for the k-th voxel.

We assume that the covariance of the multivariate Gaussian distribution from which the activity amplitudes $\boldsymbol{\beta}_k$ are generated has a scaling factor that depends on its pseudo-SNR s_k:

$$\boldsymbol{\beta}_{\cdot k} \sim \mathbf{N}(0, (s_k \sigma_k)^2 \mathbf{U}). \tag{3}$$

This is to reflect the fact that not all voxels in an ROI respond to tasks.

We further use Cholesky decomposition to parametrize the covariance structure U: $U = LL^T$, where L is a lower triangular matrix. Thus, $\boldsymbol{\beta}_{\cdot k}$ can be written as $\boldsymbol{\beta}_{\cdot k} = s_k \sigma_k L \boldsymbol{\alpha}_k$, where $\boldsymbol{\alpha}_k \sim N(0, I)$. This change of parameter allows for estimating $\mathbf{U}$ of lower rank (if the researcher has sufficient reason to make such a guess) by setting L as lower-triangular matrix with a few rightmost-columns truncated. With an improper uniform prior for $\beta_{0 \cdot k}$, and temporarily assuming X_0 is given, we have the unmarginalized likelihood for each voxel

k:

$$
\begin{aligned}
p(Y_k, &\beta_{\cdot k}, \beta_{0\cdot k}|X, X_0, L, \sigma_k, \rho_k, s_k) \\
=&p(Y_k|\beta_{\cdot k}, \beta_{0\cdot k}, X, X_0, \sigma_k, \rho_k)p(\beta_{\cdot k}|L, \sigma_k, s_k)p(\beta_{0\cdot k}) \\
=&p(Y_k|s_k\sigma_k L\alpha_k, \beta_{0\cdot k}, X, X_0, \sigma_k, \rho_k)p(\alpha_k)p(\beta_{0\cdot k}) \\
\propto&p(Y_k|s_k\sigma_k L\alpha_k, \beta_{0\cdot k}, X, X_0, \sigma_k, \rho_k)p(\alpha_k) \\
=&\exp[-\frac{1}{2}(Y_k - s_k\sigma_k XL\alpha_k - X_0\beta_{0\cdot k})^T\Sigma_{\epsilon_k}^{-1}(Y_k - s_k\sigma_k XL\alpha_k - X_0\beta_{0\cdot k})] \\
&\cdot (2\pi)^{-\frac{n_T}{2}}|\Sigma_{\epsilon_k}^{-1}|^{\frac{1}{2}}(2\pi)^{-\frac{r}{2}}\exp[-\frac{1}{2}\alpha_k^T\alpha_k]
\end{aligned}
\tag{4}
$$

where $r \leq n_C$ is the rank of L.

In contrast to the full model, our null model assumes

$$
\begin{aligned}
p(Y_k, &\beta_{0\cdot k}|X_0, \sigma_k, \rho_k) \\
=&p(Y_k|\beta_{0\cdot k}, X_0, \sigma_k, \rho_k)p(\beta_{0\cdot k}) \\
\propto&p(Y_k|\beta_{0\cdot k}, X_0, \sigma_k, \rho_k) \\
=&\exp[-\frac{1}{2}(Y_k - X_0\beta_{0\cdot k})^T\Sigma_{\epsilon_k}^{-1}(Y_k - X_0\beta_{0\cdot k})] \\
&\cdot (2\pi)^{-\frac{n_T}{2}}|\Sigma_{\epsilon_k}^{-1}|^{\frac{1}{2}}
\end{aligned}
\tag{5}
$$

For data within one run, $\Sigma_{\epsilon_k}^{-1}$, the inverse matrix of the covariance of ϵ_k, is a banded symmetric matrix which can be written as $\Sigma_{\epsilon_k}^{-1} = \frac{1}{\sigma_k^2}(I - \rho_k F + \rho_k^2 D)$, where F is 1 only at the superdiagonal and subdiagonal elements and 0 everywhere else, and D is 1 on all diagonal elements except for the first and last one, and 0 elsewhere. For abbreviation, we can denote $A_k = A(\rho_k) = I - \rho_k F + \rho_k^2 D$ which is a function of ρ_k. $\Sigma_{\epsilon_k}^{-1}$ can be factorized as $\Sigma_{\epsilon_k}^{-1} = \frac{1}{\sigma_k^2}A_k$. When Y_k includes concatenated time series across several runs, $\Sigma_{\epsilon_k}^{-1}$ is a block diagonal matrix with each block diagonal elements corresponding to one run, constructed in the same way.

To derive the log likelihood of L for data of all voxels in the ROI, we need to marginalizing all other unknown parameters. Below, we marginalize them step by step.

By marginalizing $\beta_{0\cdot k}$, we have

$$
\begin{aligned}
&p(Y_k,\beta_{\cdot k}|X, X_0, L, \sigma_k, \rho_k, s_k) \\
&\quad\propto \int p(Y_k|s_k\sigma_k L\alpha_k, \beta_{0\cdot k}, X, X_0, \sigma_k, \rho_k)p(\alpha_k)d\beta_{0\cdot k} \\
&\quad=(2\pi)^{-\frac{n_T+r-n_0}{2}}|\Sigma_{\epsilon_k}^{-1}|^{\frac{1}{2}}|X_0^T\Sigma_{\epsilon_k}^{-1}X_0|^{-\frac{1}{2}}\exp[-\frac{1}{2}\alpha_i^T\alpha_i] \\
&\quad\quad\cdot\exp[-\frac{1}{2\sigma_k^2}(Y_k - s_k\sigma_k XL\alpha_k)^T A_k^*(Y_k - s_k\sigma_k XL\alpha_k)]
\end{aligned}
\tag{6}
$$

n_0 is the number of components in X_0. In the equation above, we denoted $A_k^* = \sigma_k^2(\Sigma_{\epsilon_k}^{-1} - \Sigma_{\epsilon_k}^{-1}X_0(X_0^T\Sigma_{\epsilon_k}^{-1}X_0)^{-1}X_0^T\Sigma_{\epsilon_k}^{-1}) = A_k - A_kX_0(X_0^TA_kX_0)^{-1}X_0^TA_k$.

By further marginalizing α_k which is equivalent to marginalizing $\beta_{\cdot k}$, we get

$$
\begin{aligned}
&p(Y_k|X, X_0, L, \sigma_k, \rho_k, s_k) \\
&\quad=\int p(Y_k|s_k\sigma_k L\alpha_k, X, X_0, \sigma_k, \rho_k)p(\alpha_k)d\alpha_k \\
&\quad\propto(2\pi)^{-\frac{n_T-n_0}{2}}|\Sigma_{\epsilon_k}^{-1}|^{\frac{1}{2}}|X_0^T\Sigma_{\epsilon_k}^{-1}X_0|^{-\frac{1}{2}}|\Lambda_k^*|^{\frac{1}{2}} \\
&\quad\quad\cdot\exp[-\frac{1}{2}(\frac{1}{\sigma_k^2}Y_k^TA_k^*Y_k - \mu_k^{*T}\Lambda_k^{*-1}\mu_k^*)]
\end{aligned}
\tag{7}
$$

where $\Lambda_k^* = (I + s_k^2L^TX^TA_k^*XL)^{-1}$ and $\mu_k = \frac{s_k}{\sigma_k}\Lambda_k^*L^TX^TA_k^*Y_k$ are the variance and mean of the posterior distribution of α_k, respectively.

All the steps of marginalization above utilize the property of multivariate Gaussian distribution. Next we marginalize the noise variance σ_k^2. We assume an improper uniform distribution of σ_k^2 in $\mathbb{R}^+$. It is also possible to assume a conjugate prior for σ_k^2. Given that data of at least hundreds of time points are obtained in each run to provide enough constraint to σ_k^2, our choice does not appear to cause problem. To isolate σ_k^2, using the property

of Cholesky decomposition of $\Sigma_{\epsilon_k}^{-1}$, the above equation can be written as

$$
\begin{aligned}
&p(Y_k|X, X_0, L, \sigma_k, \rho_k, s_k) \\
&\quad \propto (2\pi)^{-\frac{n_T-n_0}{2}} \sigma_k^{2^{-\frac{n_T-n_0}{2}}} (1-\rho_k^2)^{\frac{n_r}{2}} |X_0^T A_k X_0|^{-\frac{1}{2}} |\Lambda_k^*|^{\frac{1}{2}} \\
&\qquad \cdot \exp[\frac{1}{2\sigma_k^2}(s_k^2 Y_k^T A_k^* X L \Lambda_k^* L^T X^T A_k^* Y_k - Y_k^T A_k^* Y_k)]
\end{aligned}
\tag{8}
$$

This form is proportional to an inverse-Gamma distribution of σ_k^2. n_r is the number of runs in the data. Therefore, we can analytically marginalize σ_k^2 and obtain

$$
\begin{aligned}
&p(Y_k|X, X_0, L, \rho_k, s_k) \\
&\quad = \int p(Y_k|X, X_0, L, \sigma_k, \rho_k, s_k) p(\sigma_k^2) d\sigma_k^2 \\
&\quad \propto (2\pi)^{-\frac{n_T-n_0}{2}} (1-\rho_k^2)^{\frac{n_r}{2}} |X_0^T A_k X_0|^{-\frac{1}{2}} |\Lambda_k^*|^{\frac{1}{2}} \Gamma(\frac{n_T-n_0}{2}-1) \\
&\qquad \cdot [\frac{Y_k^T A_k^* Y_k - s_k^2 Y_k^T A_k^* X L \Lambda_k^* L^T X^T A_k^* Y_k}{2}]^{1-\frac{n_T-n_0}{2}}
\end{aligned}
\tag{9}
$$

We did not find ways to further analytically marginalize s_k or ρ_k. But we can numerically marginalize them by weighted sum of (9) at $n_l \times n_m$ discrete grids $\{\rho_{kl}, s_{km}\}$ ($0 < l < n_l$, $0 < m < n_m$) with each grid representing one area of the parameter space of (ρ, s).

$$
\begin{aligned}
&p(Y_k|X, X_0, L) \\
&\quad \approx \sum_{l=1}^{n_l} \sum_{m=1}^{n_m} p(Y_k|X, X_0, L, \rho_{kl}, s_{km}) w(\rho_{kl}, s_{km}) \\
&\quad \propto \sum_{l=1}^{n_l} \sum_{m=1}^{n_m} (2\pi)^{-\frac{n_T-n_0}{2}} (1-\rho_{kl}^2)^{\frac{n_r}{2}} |X_0^T A_{kl} X_0|^{-\frac{1}{2}} |\Lambda_{klm}^*|^{\frac{1}{2}} \Gamma(\frac{n_T-n_0}{2}-1) \\
&\qquad \cdot [\frac{Y_k^T A_{kl}^* Y_k - s_{km}^2 Y_k^T A_{kl}^* X L \Lambda_{klm}^* L^T X^T A_{kl}^* Y_k}{2}]^{1-\frac{n_T-n_0}{2}} w(\rho_{kl}, s_{km})
\end{aligned}
\tag{10}
$$

The weights $w(\rho_{kl}, s_{km})$ are the prior probabilities of the two parameters in the area represented by $\{\rho_{kl}, s_{km}\}$. We assume uniform prior of ρ in (-1,1). All the simulations in

this paper used an exponential distribution as prior for s. The grids s_{km} are each chosen at the centers of mass of the prior distribution in the bins they represent in $(0, +\infty)$. All bins equally divide the area under the curve of the prior distribution for s. The implementation of our algorithm in BrainIAK includes three alternative forms of prior distributions: uniform prior in the range of (0, 1), log normal distribution approximated by the centers of mass of equally divided areas under its probability distribution (the same way as the exponential distribution), and "equal" prior which means all voxels are assumed to have a single fixed pseudo-SNR of 1.

Because we made the assumption that ϵ_k is independent across voxels. The log likelihood for all data is the sum of the log likelihood for each voxel.

$$\log p(Y|X, X_0, L) = \sum_{k=1}^{n_V} \log p(Y_k|X, X_0, L). \tag{11}$$

For the null model, the likelihood for each voxel after marginalizing β_{0i} and σ_k^2 can be similarly derived,

$$\begin{aligned} & p(Y_k|X_0, \rho_k) \\ & \quad \propto (2\pi)^{-\frac{n_T-n_0}{2}} (1-\rho_k^2)^{\frac{n_r}{2}} |X_0^T A_k X_0|^{-\frac{1}{2}} \\ & \quad \cdot \Gamma(\frac{n_T-n_0}{2}-1)[\frac{Y_k^T A_k^* Y_k}{2}]^{1-\frac{n_T-n_0}{2}} \end{aligned} \tag{12}$$

and the total log likelihood can be calculated similarly by numerically marginalizing ρ_k and summing the log likelihood for all voxels.

Part 2 Model fitting procedure

To fit the model, we need the gradient of the total log likelihood with respect to L. It can be derived that conditional on any grid of parameter pairs $\{\rho_{kl}, s_{km}\}$, the gradient of the

log likelihood for voxel k against each lower-triangular element of L is the corresponding lower-triangular element of the matrix

$$\begin{aligned}
&\frac{\partial}{\partial L}\log p(Y_k|X, X_0, L, \rho_{kl}, s_{km}) \\
&\quad = - s_{km}^2 X^T A_{kl}^* X L \Lambda_{klm}^* \\
&\quad\quad + \frac{s_{km}^2(n_T - n_0 - 2)}{Y_k^T A_{kl}^* Y_k - s_{km}^2 Y_k^T A_{kl}^* X L \Lambda_{klm}^* L^T X^T A_{kl}^* Y_k} \\
&\quad\quad \cdot (I - s_{km}^2 X^T A_{kl}^* X L \Lambda_{klm}^* L^T) X^T A_{kl}^* Y_k Y_k^T A_{kl}^* X L \Lambda_{klm}^*
\end{aligned} \tag{13}$$

where A_{kl}^* and Λ_{klm}^* are A_k^* and Λ_k^* evaluated at $\{\rho_{kl}, s_{km}\}$. The gradient of the total log likelihood against L after marginalizing over all grids $\{\rho_{kl}, s_{km}\}$ of all voxels is

$$\begin{aligned}
&\frac{\partial}{\partial L}\log p(Y|X, X_0, L) \\
&\quad = \sum_{k=1}^{n_V}\sum_{l=1}^{n_l}\sum_{m=1}^{n_m} p(\rho_{kl}, s_{km}|Y_k, X, X_0, L)\frac{\partial}{\partial L}\log p(Y_k|X, X_0, L, \rho_{kl}, s_{km})
\end{aligned} \tag{14}$$

$p(\rho_{kl}, s_{km}|Y_k, X, X_0, L)$ is the posterior probability of $\{\rho_{kl}, s_{km}\}$ conditional on a given L. It can be obtained by normalizing $p(Y_k|X, X_0, L, \rho_{kl}, s_{km})w(\rho_{kl}, s_{km})$ after calculating (9).

With the gradient in (14), the total log likelihood in (11) can be maximized using gradient-based method such as Broyden–Fletcher–Goldfarb–Shanno (BFGS) algorithm to search for the optimal L (1; 2; 3; 4).

However, the derivations above have made the assumption that X_0 is given, while it is not. The requirement for X_0 should be to appropriately capture the correlation of noise across voxels without overfitting. Therefore, at the starting of the model fitting, regular regression of Y against X and any nuisance regressors such as head motion and constant baseline is performed. Then the algorithm by Gavish and Donoho(5) is used to select the optimal number of components n_0 to choose X_0 from the eigenvectors of the residual of regression. Because regular regression does not shrink the magnitudes of β, their magnitudes can only be

over-estimated. n_0 thus has no risk of being over-estimated. This n_0 is then fixed throughout the model fitting. Next, the first n_0 principal components of the residual of regression are set as $\hat{X}_0$ to allow for calculating the marginal log likelihood in (14) and gradient ascent with BFGS. A sufficient steps of iterations are performed to optimize L. Then $\hat{\beta}_{\text{post}}$, the posterior expectations of β, are calculated with the current $\hat{L}$ and with s, ρ, σ being marginalized. $\hat{X}_0$ is subsequently recalculated using PCA from the residuals after subtracting $X\hat{\beta}_{\text{post}}$ from Y. The alternation between optimizing L and re-estimating $\hat{X}_0$ is repeated until convergence.

Once we obtain $\hat{L}$, the estimate of L, the estimate of the covariance structure is $\hat{U} = \hat{L}\hat{L}^T$. Converting it into a correlation matrix yields the similarity matrix by BRSA. Even though $\hat{X}_0$ is estimated from data based on posterior estimation of β repeatedly during fitting, L is still optimized for the log likelihood with all other unknown variables marginalized. Thus the estimated $\hat{U}$ is an empirical prior of β estimated from data. This is the reason we consider our model as an empirical Bayesian method.

Many subcomponents of the expressions in these equations do not depend on L and thus can be pre-computed before optimizing for L. The fixed grids of (ρ, s) further make several subcomponents shared across voxels when evaluating (9). These all reduce the amount of computation needed.

The fitting of the null model is similar to that of the full model except that there is no L to be optimized.

Part 3 Model selection and decoding task-related signals

Once a model has been fitted to some data from a participant or a group of participants, we can estimate the posterior mean of ρ, s, σ^2, β and β_0, conditional on the empirical prior $\hat{U}$ (essentially $\hat{L}$), data Y, design matrix X and the estimated intrinsic fluctuations $\hat{X}_0$. Below, we derive their formula and the procedure in which they are used for calculating

cross-validated log likelihood of new data and decoding task-related signal $\hat{X}_{\text{test}}$ and $\hat{X}_{0\text{test}}$ from new data in the context of fMRI decoding.

The posterior mean of these variables are

$$
\begin{aligned}
\hat{\sigma^2}_{k(\text{post})} =& \sum_{l=1}^{n_l}\sum_{m=1}^{n_m} p(\rho_{kl}, s_{km}|Y_k, X, \hat{X}_0, \hat{L}) \int \sigma_k^2 p(\sigma_k^2|Y_k, L, X, \hat{X}_0, \rho_{kl}, s_{km}) d\sigma_k \\
=& \sum_{l=1}^{n_l}\sum_{m=1}^{n_m} p(\rho_{kl}, s_{km}|Y_k, X, \hat{X}_0, \hat{L}) \frac{1}{n_T - n_0 - 4} \\
& \cdot Y_k^T A_{kl}^* Y_k - s_{km}^2 Y_k^T A_{kl}^* X \hat{L} \Lambda_{klm}^* \hat{L}^T X^T A_{kl}^* Y_k
\end{aligned}
\tag{15}
$$

$$
\hat{s}_{k(\text{post})} = \sum_{l=1}^{n_l}\sum_{m=1}^{n_m} p(\rho_{kl}, s_{km}|Y_k, X, \hat{X}_0, \hat{L}) s_{km} \tag{16}
$$

$$
\hat{\rho}_{k(\text{post})} = \sum_{l=1}^{n_l}\sum_{m=1}^{n_m} p(\rho_{kl}, s_{km}|Y_k, X, \hat{X}_0, \hat{L}) \rho_{kl} \tag{17}
$$

$$
\hat{\beta}_{\cdot k(\text{post})} = \sum_{l=1}^{n_l}\sum_{m=1}^{n_m} p(\rho_{kl}, s_{km}|Y_k, X, \hat{X}_0, \hat{L}) s_{km}^2 \hat{L} \Lambda_{klm} \hat{L}^T X^T A_{kl}^* Y_k \tag{18}
$$

$$
\begin{aligned}
\hat{\beta}_{0\cdot k(\text{post})} =& \sum_{l=1}^{n_l}\sum_{m=1}^{n_m} p(\rho_{kl}, s_{km}|Y_k, X, \hat{X}_0, \hat{L}) \\
& \cdot (\hat{X}_0^T A_{kl}^* \hat{X}_0)^{-1} \hat{X}_0^T A_{kl}^* (Y_k - s_{km}^2 X \hat{L} \Lambda_{klm} \hat{L}^T X^T A_{kl}^* Y_k)
\end{aligned}
\tag{19}
$$

For null model, $\hat{\sigma^2}_{k(\text{post})}$, $\hat{\beta}_{0\cdot k(\text{post})}$ and $\hat{\rho}_{k(\text{post})}$ are of similar forms except that all terms including s_{km} are removed and that $p(\rho_{kl}, s_{km}|Y_k, X, \hat{X}_0, \hat{L})$ is replaced by $p(\rho_{kl}|Y_k, \hat{X}_0)$.

To calculate cross-validated log likelihood, we assume the posterior estimates above and the statistical properties of X_0 stay unchanged in the testing data. We use zero-mean AR(1)

process to describe the statistical properties of X_0. The AR(1) parameters estimated from $\hat{X}_0$ serve as the parameters of the empirical prior for X_0 in the testing data. When X_0 at each time point t is treated as a random vector $X_0^{(t)}$, the AR(1) parameters of each component can be jointly written as the diagonal matrix $V_{\Delta X_0}$ for the variance of the innovation noise, and diagonal matrix T_{X_0} for the auto-regressive coefficients, both of size $n_0 \times n_0$.

For model selection purpose, design matrix X_{test} for the testing data should be generated in the same manner as they are for the training data by the researcher. For full BRSA model, $X_{\text{test}}\hat{\beta}_{\text{post}}$ is the predicted task-related signal in Y_{test}. $Y_{\text{res}} = Y_{\text{test}} - X_{\text{test}}\hat{\beta}_{\text{post}}$ is the residual variation which cannot be explained by the design matrix and the posterior activity pattern $\hat{\beta}_{\text{post}}$. Null model does not predict any task-related activity, so all Y_{test} constitutes residual variation Y_{res}. In either the full model or the null model, the posterior estimate $\hat{\beta}_{0(\text{post})}$ expresses their prediction about how voxels should be co-modulated by a fluctuation, while the fluctuation time course $X_{0\text{test}}$ is only predictable in terms of its variance and temporal autocorrelation expressed by $V_{\Delta X_0}$ and T_{X_0}. $\hat{\sigma^2}_{k(\text{post})}$ and $\hat{\rho}_{k(\text{post})}$ express the models' predictions about the variance and temporal dependency of the fluctuation in the k-th voxel in addition to the co-fluctuation. With these parameters estimated from training data, both the full and null models can marginalize the unknown $X_{0\text{test}}$ and yield their corresponding predictive log likelihoods for the testing data Y_{test}. These log likelihoods are the basis for selecting between the full and null models.

To calculate the log likelihood, we notice that the predictive model of Y_{res} in the testing data by both models are dynamical system models in which $X_{0\text{test}}$ is the latent state and Y_{res} is the observed data. They are slightly different from the standard dynamical system model(6) in that not only the latent states, but also the noise, have temporal dependency(7):

$$X_{0\text{test}}^{(t)} \sim N(X_{0\text{test}}^{(t-1)} T_{X_0}, V_{\Delta X_0}) \tag{20}$$

$$
\begin{aligned}
&Y_{\text{res}}^{(t)} - X_{0\text{test}}^{(t)}\hat{\beta}_{0\text{post}} \\
&\quad \sim N((Y_{\text{res}}^{(t)} - X_{0\text{test}}^{(t)}\hat{\beta}_{0\text{post}})\text{Diag}(\hat{\rho}_{\text{post}}), \text{Diag}(\hat{\sigma^2}_{\text{post}}))
\end{aligned} \tag{21}
$$

Where $\text{Diag}(\hat{\rho}_{\text{post}})$ and $\text{Diag}(\hat{\sigma^2}_{\text{post}})$ are diagonal matrices with vectors $\hat{\rho}_{\text{post}}$ and $\hat{\sigma^2}_{\text{post}}$ being their diagonal elements, respectively.

Because a modified forward-backward algorithm from the standard approach(6) is needed to calculate the preditive log likelihood $p(Y_{\text{res}}|\hat{\beta}_{0\text{post}}, T_{X_0}, V_{\Delta X_0}, \text{Diag}(\hat{\rho}_{\text{post}}), \text{Diag}(\hat{\sigma^2}_{\text{post}}))$ and the posterior distribution of $X_{0\text{test}}$, we describe the procedure below.

Define

$$
\hat{G}(X_{0\text{test}}^{(t)}) = p(X_{0\text{test}}^{(t)}|Y_{\text{res}}^{(1)}, \cdots, Y_{\text{res}}^{(t)}) \tag{22}
$$

$$
\hat{H}(X_{0\text{test}}^{(t)}) = \frac{p(Y_{\text{res}}^{(t+1)}, \cdots, Y_{\text{res}}^{(n_T)}|X_{0\text{test}}^{(t)}, Y_{\text{res}}^{(t)})}{p(Y_{\text{res}}^{(t+1)}, \cdots, Y_{\text{res}}^{(n_T)}|Y_{\text{res}}^{(1)}, \cdots, Y_{\text{res}}^{(t)})}, \text{ for } t < n_T \tag{23}
$$

and

$$
\begin{aligned}
&c_t = p(Y_{\text{res}}^{(t)}|Y_{\text{res}}^{(1)}, \cdots, Y_{\text{res}}^{(t-1)}), \text{ for } t > 0 \\
&c_1 = p(Y_{\text{res}}^{(1)})
\end{aligned} \tag{24}
$$

Therefore, the cross-validated log likelihood is

$$
\log p(Y_{\text{res}}^{(1)}, \cdots, Y_{\text{res}}^{(n_T)}|\hat{\beta}_{0\text{post}}, T_{X_0}, V_{\Delta X_0}, \hat{\sigma^2}_{\text{post}}, \hat{\rho}_{\text{post}}) = \sum_{t=1}^{n_T} \log c_t \tag{25}
$$

It can be derived that the posterior distribution of $X_{0\text{test}}^{(t)}$ is

$$
\gamma(X_{0\text{test}}^{(t)}) = p(X_{0\text{test}}^{(t)}|Y_{\text{res}}^{(1)}, \cdots, Y_{\text{res}}^{(n_T)}) = \hat{G}(X_{0\text{test}}^{(t)})\hat{H}(X_{0\text{test}}^{(t)}) \tag{26}
$$

Below, we denote the mean and covariance of $\hat{G}(X_{0\text{test}}^{(t)})$ as $\mu_{X_0}^{(t)}$ and $\Gamma_{X_0}^{(t)}$, and the mean and covariance of $\gamma(X_{0\text{test}}^{(t)})$ as $\tilde{\mu}_{X_0}^{(t)}$ and $\tilde{\Gamma}_{X_0}^{(t)}$.

$\mu_{X_0}^{(t)}$, $\Gamma_{X_0}^{(t)}$ and c_t can be calculated by the forward step. $\tilde{\mu}_{X_0}^{(t)}$ and $\tilde{\Gamma}_{X_0}^{(t)}$ can be calculated by the backward step. To perform model selection, only forward step is necessary.

To perform the forward step, we first note that for $t = 1$

$$X_{0\text{test}}^{(1)} \sim N(0, V_{\Delta X_0}(I - T_{X_0}^2)^{-1}) \tag{27}$$

and

$$Y_{\text{res}}^{(1)} \sim N(X_{0\text{test}}^{(1)}\hat{\beta}_{0\text{post}}, \text{Diag}(\hat{\sigma^2}_{\text{post}})(I - \text{Diag}(\hat{\rho^2}_{\text{post}}))^{-1}) \tag{28}$$

Denote $V_{X_0} = V_{\Delta X_0}(I - T_{X_0}^2)^{-1}$, we have

$$\begin{aligned}
c_1\hat{G}(X_{0\text{test}}^{(1)}) &= p(X_{0\text{test}}^{(1)}|Y_{\text{res}}^{(1)})p(Y_{\text{res}}^{(1)}) \\
&= p(X_{0\text{test}}^{(1)})p(Y_{\text{res}}^{(1)}|X_{0\text{test}}^{(1)}) \\
&= (2\pi)^{-\frac{n_0}{2}}|V_{X_0}|^{-\frac{1}{2}}\exp[-\frac{1}{2}X_{0\text{test}}^{(1)}V_{X_0}^{-1}X_{0\text{test}}^{(1)\,T}] \\
&\cdot \exp[-\frac{1}{2}\sum_{k=1}^{n_V}\frac{(Y_{k\text{res}}^{(1)} - X_{0\text{test}}^{(1)}\hat{\beta}_{0\cdot k\text{post}})^2(1-\rho_{k(\text{post})}^2)}{\sigma_{k(\text{post})}^2}]\prod_{k=1}^{n_V}(\frac{1-\rho_{k(\text{post})}^2}{\sigma_{k(\text{post})}^2})^{\frac{1}{2}}
\end{aligned} \tag{29}$$

$\hat{G}(X_{0\text{test}}^{(1)})$ is a multivariate normal distribution of $X_{0\text{test}}^{(1)}$, we can find its covariance and mean from (29):

$$\Gamma_{X_0}^{(1)} = [V_{X_0}^{-1} + \hat{\beta}_{0\text{post}}(I - \text{Diag}(\hat{\rho^2}_{\text{post}}))\text{Diag}(\hat{\sigma^2}_{\text{post}})^{-1}\hat{\beta}_{0\text{post}}^T]^{-1} \tag{30}$$

$$\mu_{X_0}^{(1)} = Y_{\text{res}}^{(1)}(I - \text{Diag}(\hat{\rho^2}_{\text{post}}))\text{Diag}(\hat{\sigma^2}_{\text{post}})^{-1}\hat{\beta}_{0\text{post}}^T\Gamma_{X_0}^{(1)} \tag{31}$$

Because $\hat{G}(X_{0\text{test}}^{(1)})$ is a normalized probability distribution, the components in (29) after

factoring out the multivariate normal distribution $\hat{G}(X^{(1)}_{0\text{test}})$ is c_1:

$$\begin{aligned} c_1 =&(2\pi)^{-\frac{n_V}{2}}|V_{X_0}|^{-\frac{1}{2}}|\Gamma^{(1)}_{X_0}|^{\frac{1}{2}}\prod_{k=1}^{n_V}(\frac{\hat{\sigma^2}_{k(\text{post})}}{1-\hat{\rho^2}_{k(\text{post})}})^{-\frac{1}{2}} \\ &\cdot \exp\{-\frac{1}{2}[Y^{(1)}_{\text{res}}(I-\text{Diag}(\hat{\rho^2}_{\text{post}}))\text{Diag}(\hat{\sigma^2}_{\text{post}})^{-1}{Y^{(1)}_{\text{res}}}^T \\ &-\mu^{(1)}_{X_0}{\Gamma^{(1)}_{X_0}}^{-1}{\mu^{(1)}_{X_0}}^T]\} \end{aligned} \tag{32}$$

For any $t > 1$, the following relation holds:

$$\begin{aligned} &c_t\hat{G}(X^{(t)}_{0\text{test}}) \\ &=\int p(Y^{(t)}_{\text{res}}|X^{(t)}_{0\text{test}}, X^{(t-1)}_{0\text{test}}, Y^{(t-1)}_{\text{res}})p(X^{(t)}_{0\text{test}}|X^{(t-1)}_{0\text{test}})\hat{G}(X^{(t-1)}_{0\text{test}})dX^{(t-1)}_{0\text{test}} \end{aligned} \tag{33}$$

$p(Y^{(t)}_{\text{res}}|X^{(t)}_{0\text{test}}, X^{(t-1)}_{0\text{test}}, Y^{(t-1)}_{\text{res}})$ is defined by (21). $p(X^{(t)}_{0\text{test}}|X^{(t-1)}_{0\text{test}})$ is defined by (20). Mean and covariance of $\hat{G}(X^{(t-1)}_{0\text{test}})$ are calculated by the previous step for $t-1$. Therefore, by marginalizing $X^{(t-1)}_{0\text{test}}$, we obtain

$$\Gamma^{(t)}_{X_0} = (K_2 - J(K_1 + {\Gamma^{(t-1)}_{X_0}}^{-1})J^T)^{-1} \tag{34}$$

and

$$\begin{aligned} \mu^{(t)}_{X_0} = [&\Delta Y^{(t)}_{\text{res}}\text{Diag}(\hat{\sigma^2}_{\text{post}})^{-1}\hat{\beta}^T_{0\text{post}} + (\mu^{(t-1)}_{X_0}{\Gamma^{(t-1)}_{X_0}}^{-1} \\ &-\Delta Y^{(t)}_{\text{res}}\text{Diag}(\hat{\sigma^2}_{\text{post}})^{-1}\text{Diag}(\hat{\rho}_{\text{post}})\hat{\beta}^T_{0\text{post}})(K_1+\Gamma^{(t-1)}_{X_0})^{-1}J^T]\Gamma^{(t)}_{X_0} \end{aligned} \tag{35}$$

where $\Delta Y^{(t)}_{\text{res}} = Y^{(t)}_{\text{res}} - Y^{(t-1)}_{\text{res}}\text{Diag}(\hat{\rho}_{\text{post}})$. $J = V^{-1}_{\Delta X_0}T^T_{X_0} + \hat{\beta}_{0\text{post}}\text{Diag}(\hat{\sigma^2}_{\text{post}})^{-1}\text{Diag}(\hat{\rho}_{\text{post}})\hat{\beta}^T_{0\text{post}}$, $K_1 = T_{X_0}V^{-1}_{\Delta X_0}T^T_{X_0} + \hat{\beta}_{0\text{post}}\text{Diag}(\hat{\sigma^2}_{\text{post}})^{-1}\text{Diag}(\hat{\rho}_{\text{post}})^2\hat{\beta}^T_{0\text{post}}$ and $K_2 = V^{-1}_{\Delta X_0} + \hat{\beta}_{0\text{post}}\text{Diag}(\hat{\sigma^2}_{\text{post}})^{-1}\hat{\beta}^T_{0\text{post}}$. Note that J, K_1, K_2 are all constants.

Similarly to (32), after factoring out $\hat{G}(X_{0\text{test}}^{(t)})$, we obtain

$$\begin{aligned}
c_t = (2\pi)^{-\frac{n_V}{2}} |K_1 + \Gamma_{X_0}^{(t-1)}|^{-\frac{1}{2}} |V_{\Delta X_0}|^{-\frac{1}{2}} |\Gamma_{X_0}^{(t-1)}|^{-\frac{1}{2}} |\Gamma_{X_0}^{(t)}|^{\frac{1}{2}} \prod_{k=1}^{n_V} \sigma_{k\text{post}}^{-1} \\
\cdot \exp[-\frac{1}{2}\mu_{X_0}^{(t-1)} \Gamma_{X_0}^{(t-1)^{-1}} \mu_{X_0}^{(t-1)^T} + \frac{1}{2}\mu_{X_0}^{(t)} \Gamma_{X_0}^{(t)^{-1}} \mu_{X_0}^{(t)^T} \\
- \frac{1}{2}\Delta Y_{\text{res}}^{(t)} \text{Diag}(\hat{\sigma^2}_{\text{post}})^{-1} \Delta Y_{\text{res}}^{(t)T} + \frac{1}{2}(\mu_{X_0}^{(t-1)} \Gamma_{X_0}^{(t-1)^{-1}} \\
- \Delta Y_{\text{res}}^{(t)} \text{Diag}(\hat{\sigma^2}_{\text{post}})^{-1} \text{Diag}(\hat{\rho}_{\text{post}}) \hat{\beta}_{0\text{post}}^T)(K_1 + \Gamma_{X_0}^{(t-1)})^{-1} \\
(\mu_{X_0}^{(t-1)} \Gamma_{X_0}^{(t-1)^{-1}} - \Delta Y_{\text{res}}^{(t)} \text{Diag}(\hat{\sigma^2}_{\text{post}})^{-1} \text{Diag}(\hat{\rho}_{\text{post}}) \hat{\beta}_{0\text{post}}^T)^T]
\end{aligned} \tag{36}$$

By calculating (34), (35) and (36) recursively with t incremented by 1 until n_T, the predictive log likelihood (25) of both the full and null models can be calculated to serve as the basis of model selection.

To calculate the mean and variance of the posterior distribution $\gamma({X_{0\text{test}}}^{(t)})$ of $X_{0\text{test}}$, backward step is needed. We denote its mean as $\hat{\mu}_{X_0}^{(t)}$, and covariance as $\hat{\Gamma}_{X_0}^{(t)}$.

For any $t < n_T$, it can be derived that

$$\begin{aligned}
c_{t+1}\hat{H}(X_{0\text{test}}^{(t)}) = \\
\int \hat{H}(X_{0\text{test}}^{(t+1)}) p(X_{0\text{test}}^{(t+1)} | X_{0\text{test}}^{(t)}) p(Y_{\text{res}}^{(t+1)} | X_{0\text{test}}^{(t)}, X_{0\text{test}}^{(t+1)}, Y_{\text{res}}^{(t)}) dX_{0\text{test}}^{(t+1)}
\end{aligned} \tag{37}$$

By plugging in (26), we get

$$\begin{aligned}
\gamma({X_{0\text{test}}}^{(t)}) = \frac{\hat{G}(X_{0\text{test}}^{(t)})}{c_{t+1}} \\
\cdot \int \frac{\gamma({X_{0\text{test}}}^{(t+1)})}{\hat{G}(X_{0\text{test}}^{(t+1)})} p(X_{0\text{test}}^{(t+1)} | X_{0\text{test}}^{(t)}) p(Y_{\text{res}}^{(t+1)} | X_{0\text{test}}^{(t)}, X_{0\text{test}}^{(t+1)}, Y_{\text{res}}^{(t)}) dX_{0\text{test}}^{(t+1)}
\end{aligned} \tag{38}$$

After the marginalization in (38) and observing the terms related to ${X_{0\text{test}}}^{(t)}$, we get the

recursive relations

$$\hat{\Gamma}_{X_0}^{(t)} = ({\Gamma_{X_0}^{(t)}}^{-1} + K_1 - J^T({\hat{\Gamma}_{X_0}^{(t+1)}}^{-1} - {\Gamma_{X_0}^{(t+1)}}^{-1} + K_2)^{-1} J)^{-1} \tag{39}$$

and

$$\begin{aligned}\hat{\mu}_{X_0}^{(t)} = &[\mu_{X_0}^{(t)}{\Gamma_{X_0}^{(t)}}^{-1} - \Delta Y_{\text{res}}^{(t+1)}\text{Diag}(\hat{\sigma^2}_{\text{post}})^{-1}\text{Diag}(\hat{\rho}_{\text{post}})\hat{\beta}_{0\text{post}}^T \\ &+ (\hat{\mu}_{X_0}^{(t+1)}{\hat{\Gamma}_{X_0}^{(t+1)}}^{-1} - \mu_{X_0}^{(t+1)}{\Gamma_{X_0}^{(t+1)}}^{-1} + \Delta Y_{\text{res}}^{(t+1)}\text{Diag}(\hat{\sigma^2}_{\text{post}})^{-1}\hat{\beta}_{0\text{post}}^T) \\ &({\hat{\Gamma}_{X_0}^{(t+1)}}^{-1} - {\Gamma_{X_0}^{(t+1)}}^{-1} + K_2)^{-1}J]\hat{\Gamma}_{X_0}^{(t)}\end{aligned} \tag{40}$$

Note that $\gamma({X_{0\text{test}}}^{(n_T)}) = \hat{G}(X_{0\text{test}}^{(n_T)})$, therefore $\hat{\mu}_{X_0}^{(n_T)} = \mu_{X_0}^{(n_T)}$ and $\hat{\Gamma}_{X_0}^{(n_T)} = \Gamma_{X_0}^{(n_T)}$. By recursively calculating (39) and (40) with t decremented by 1 from $n_T - 1$ until 1, the posterior distribution of $X_{0\text{test}}^{(t)}$ given all the testing data can be calculated.

For decoding purpose, we need to obtain not only the posterior mean of intrinsic fluctuations $X_{0\text{test}}^{(t)}$, but also the task-related activity $X_{\text{test}}^{(t)}$. Therefore, we do not subtract a predicted signal $X_{\text{test}}\hat{\beta}_{\text{post}}$ based on a hypothetical design matrix from testing data Y_{test}. We perform the forward-backward algorithm on Y_{test} directly. By replacing $\hat{\beta}_{0\text{post}}$ in the equations from (20) to (40) with $[\hat{\beta}_{\text{post}}^T, \hat{\beta}_{0\text{post}}^T]^T$ and other related terms accordingly, the posterior mean of both $X_{\text{test}}^{(t)}$ and $X_{0\text{test}}^{(t)}$ can be decoded just as $X_{0\text{test}}^{(t)}$ is decoded in (40).

Part 4 Performance of all methods when all voxels have task-related signals

In the main article, we consider the scenario when not all voxels in a selected ROI respond to the task of interest. Here we test the performance of BRSA with a simulation when all voxels respond to a task with equal SNR. The results are displayed in in Figure **1** of **S1**

Materials. The simulation procedure was mostly the same as in Figure **3** of the main article. The only difference is that $\hat{\beta}$ were sampled according to the covariance matrix in Figure **3A** of the main article for all the voxels in the ROI (Figure **3B** of the main article), and then multiplied with values in $\{0.0625, 0.125, 0.25, 0.1\}$ times the standard deviation of the detrended noise (resting state data) in each voxel. The resulting average SNRs across voxels are $\{0.017, 0.034, 0.068, 0.361\}$, respectively. Uniform prior of pseudo-SNR was used in BRSA (although see next section that the choice of the prior has no impact). Repeated-measures ANOVA on results with 2 and 4 runs of data indicates significant main effects of RSA methods (F=178.1, p<2e-42), of amounts of data (F=53.2, p<2e-7) and of SNR levels (F=1225.8, p<9e-60). There are also significant interactions between RSA methods and amounts of data (F=4.7, p<1.6e-3), between RSA methods and SNR levels (F=96.2, p<4e-91) and between amounts of data and SNR levels (F=244.2, p<1e-36), and significant interaction among all three factors (F=44.4, p<2e-57). Post-hoc paired t-test between RSA methods show no significant difference between BRSA and cross-run RSA with spatial whitening (t=-1.3, p=0.19). Both BRSA and cross-run RSA with spatial whitening are significantly better than all other methods (largest p=1.2e-10). Further repeated-measures ANOVA between BRSA and cross-run RSA with spatial whitening shows significant interaction between methods and amounts of data (F=4.6, p<0.04), significant interaction between methods and SNR (F=7.0, p<4e-4) and a significant interaction among three factors (F=18.7, p<6e-9). These results show that in cases where all voxels in an ROI have homogeneous SNR, the average performance of BRSA is indistinguishable from cross-run RSA with spatial whitening, but still better than all other approaches including traditional within-run RSA.

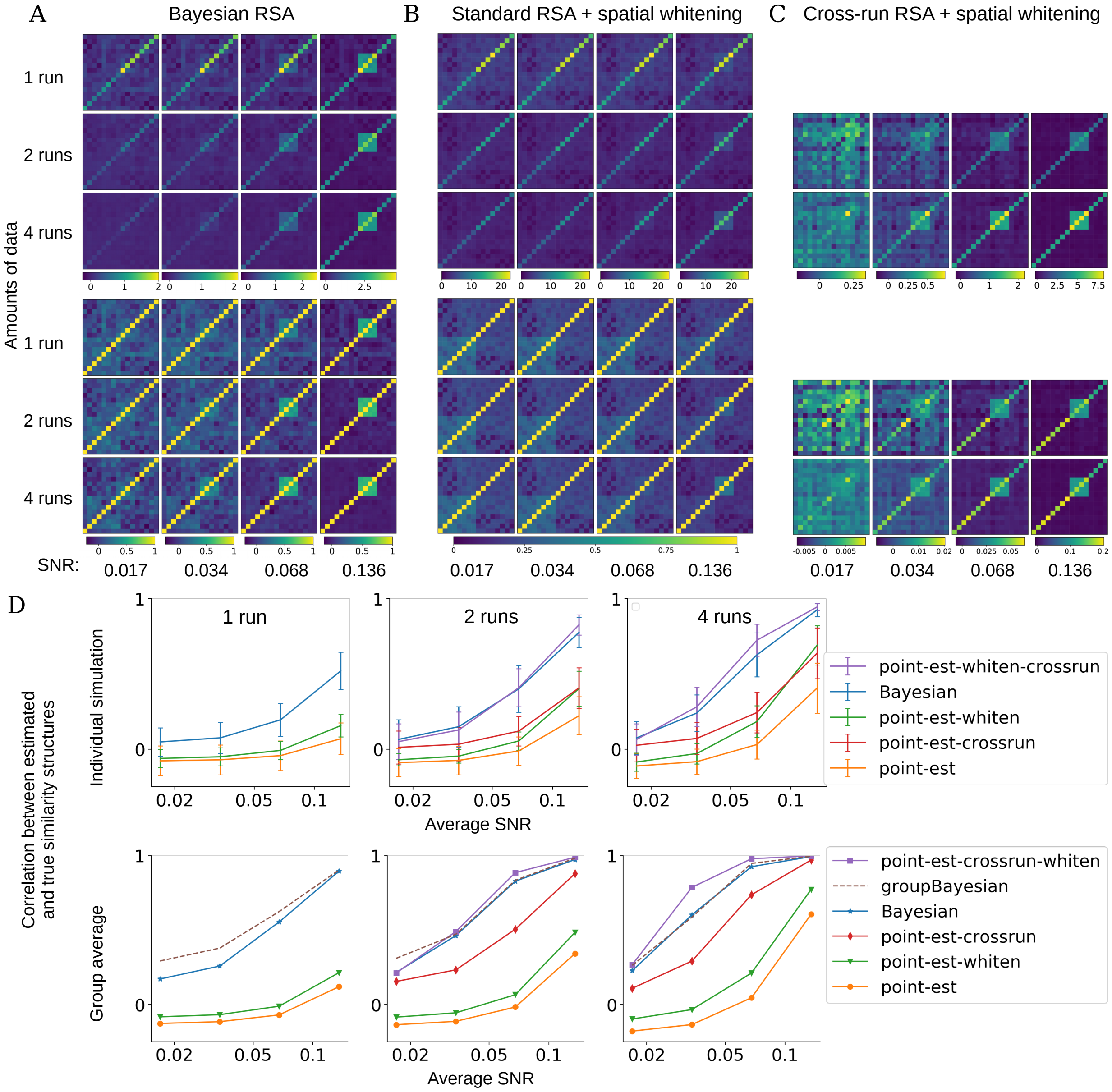

Figure 1: **Performance of BRSA and other methods when all voxels in an ROI respond to task conditions.** We used the same "noise" in simulation as in Figure 3 of the main article, i.e., from the same ROI of the resting state fMRI data from HCP. The difference is that task-related activity amplitudes were sampled for all voxels in the simulated ROI, and lower SNRs were used. **(A)** Average covariance matrix (top) and similarity matrix (bottom) estimated by BRSA, across different SNR levels (columns) and different numbers of runs (rows). The average SNRs over the whole ROI are displayed at the bottom.

Figure 1: **(B)** The corresponding result obtained by standard RSA based on activity patterns estimated within runs, which are spatially whitened. **(C)** The corresponding result of RSA based on cross-correlating patterns estimated from separate runs, which are spatially whitened based on the residuals of all scanning runs. **(D)** Top: average correlation (mean $\pm$ std) between the off-diagonal elements of the estimated and true similarity matrices, for each method, across SNR levels (x-axis) and amounts of data (separate plots). Bottom: The correlation between the average estimated similarity matrix of each method and the true similarity matrix.

Part 5 Comparison of BRSA with different assumptions of the prior of pseudo-SNR

As many Bayesian models, certain choices of the form of the prior distributions of some parameters need to be made. In BRSR model, we implemented four types of prior distributions for the pseudo-SNR: exponential distribution, uniform distribution, log-normal distribution and Delta distribution (This distribution assumes pseudo-SNR s=1 for all voxels. We denote it as "equal" assumption since it indicates all voxels have equal SNR). We believe these reflect the common shapes of distributions one may hypothesize about the level of SNR within an ROI. Here we investigate the degree by which the choice of prior distribution influence the performance of BRSA. We performed the same simulation as in Figure **3** of the main article, but fitted BRSA model separately using each of these four types of priors. As shown in Figure **2A** of **S1** Material, in this simulation where a subset of voxels contained task-related responses, overall log normal prior performed the best and "equal" prior performed the worst. Repeated-measures ANOVA indicate significant main effects of the form of SNR prior (F=261.4, p<1e-37), amount of data (F=47.3, p<5e-7) and SNR level (F=594.3, p<3e-49), and significant interactions between SNR prior and amounts of data (F=28.6, p<4e-12), between SNR prior and SNR level (F=79.6, p<3e-62) and between amounts of data and SNR level (F=11.4, p<4e-6), and a significant interaction among the three factors (F=44.3, p<1e-43). Since we are mainly interested in the effect of the form of SNR prior, post-hoc paired

t-tests were performed among them. There were significant difference between every pairs of the comparison (the largest p=0.0015 between log normal and exponential distributions) and the performance was log normal > exponential > uniform > "equal". This order was also consistent with the observation that the fitted pseudo-SNRs were the most separate between task-active and task-inactive voxels when assuming log normal prior (Figure **2C** of **S1** Materials). However, under all the three forms of prior distributions that allow variation of SNR across voxels, correlations between the fitted pseudo-SNR and the empirical SNR calculated as $\frac{\sigma(\mathbf{X}\beta)}{\sigma(\text{noise})}$ (Figure **2B** of **S1** Material) were significant. When calculated within voxels with task-related signals, r=0.62, 0.62 and 0.56, for log normal, exponential and uniform prior, respectively, with the largest p=4e-16. When calculated over all simulated voxels, r=0.84, 0.82 and 0.57, with p=0.

When the same comparison was made on the simulated data in Figure **1** of **S1** Materials, in which all voxels had signals added, there was no difference between any of the form of prior distributions of pseudo-SNR, as shown in Figure **2D** of **S1** Materials. Repeated-measures ANOVA shows no main effect of the form of SNR priors (F=2.3, p=0.09), no interaction between SNR prior and amounts of data (F=0.5, p=0.7), and no interaction between SNR prior and SNR level (F=1.8, p=0.07).

These analyses suggest that when the SNR is almost equal in an ROI, the performance of BRSA is robust regardless of the choice of the form of the prior distribution of pseudo-SNR. However, in cases when SNR varies in an ROI, choosing a proper form of prior can improve its performance. This clearly demonstrates the advantage of allowing various form of priors on pseudo-SNR.

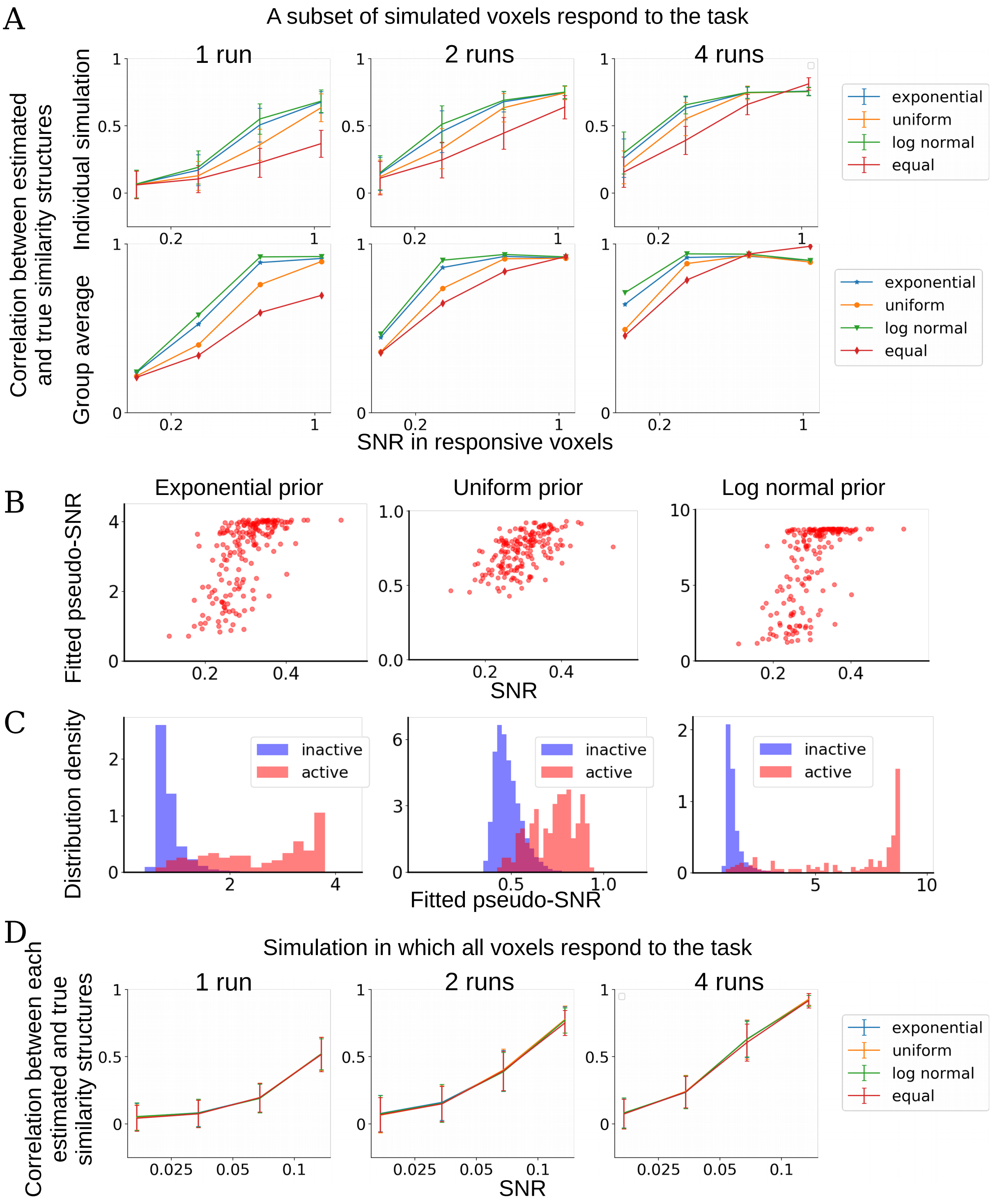

Figure 2: **The impact of the choice of SNR on the performance of BRSA. (A)** We used the same simulated data as in Figure **3** of the main article and fitted BRSA models assuming different assumptions of the form of prior distribution of pseudo-SNR: exponential, uniform, log normal and "equal" (Delta distribution).

Figure 2: **(A)** Top: the correlation (mean $\pm$ std) between off-diagonal elements of each estimated similarity matrix and the true similarity matrix. Bottom: Correlation between the average estimated similarity over the 24 simulated subjects with the true simulated one. **(B)** Scatter plot of the estimated pseudo-SNR of each voxel with added task-related signals and their corresponding empirical SNR estimated post-hoc. **(C)** The density of the distributions of the fitted pseudo-SNRs of voxels with task-related signals added. **(D)** The same analysis was performed on the simulated data in Figure **1** of **S1** Materials, where all voxels were added with simulated task-related responses. The correlation between the off-diagonal elements of the estimated similarity matrix with those of the true similarity matrix.

Part 6 The effect of the number of nuisance regressors on BRSA performance

To capture the spatial noise correlation, BRSA relies on marginalizing the amplitudes of modulation β_0 in each voxel by a set of shared time courses of intrinsic fluctuation $\mathbf{X}_0$ (nuisance regressors). $\mathbf{X}_0$ in turn needs to be estimated as the first few principal components of the residual after removing the posterior estimates of task-related activity by BRSA during its iterative fitting procedure (see *Model fitting procedure* above). We used the algorithm proposed in (5) to estimate the optimal number of principal components to be extracted as nuisance regressors. Here we evaluate the performance of BRSA when using the number of components selected by this algorithm, compared to the performance when choosing a fixed number of components from a set: $\{0, 10, 20, 40, 60\}$. The simulation setting is exactly the same as in Figure **3** of the main article. The automatically determined numbers of nuisance regressors was 6.7$\pm$1.9, 36.8$\pm$18.3 and 85.3$\pm$23.0 (mean$\pm$std) when fitted to 1, 2 and 4 runs of data, respectively, independent of variation in SNR levels. As shown from Figure **3** of **S1** Materials, the performance degrades when no nuisance regressors was used, but is generally similar across the choices of the number of nuisance regressors. Adding more nuisance regressors beyond that chosen by the algorithm (5) does not further improve performance. In fact, with small amount of data (1 run), including larger numbers of nuisance regressors

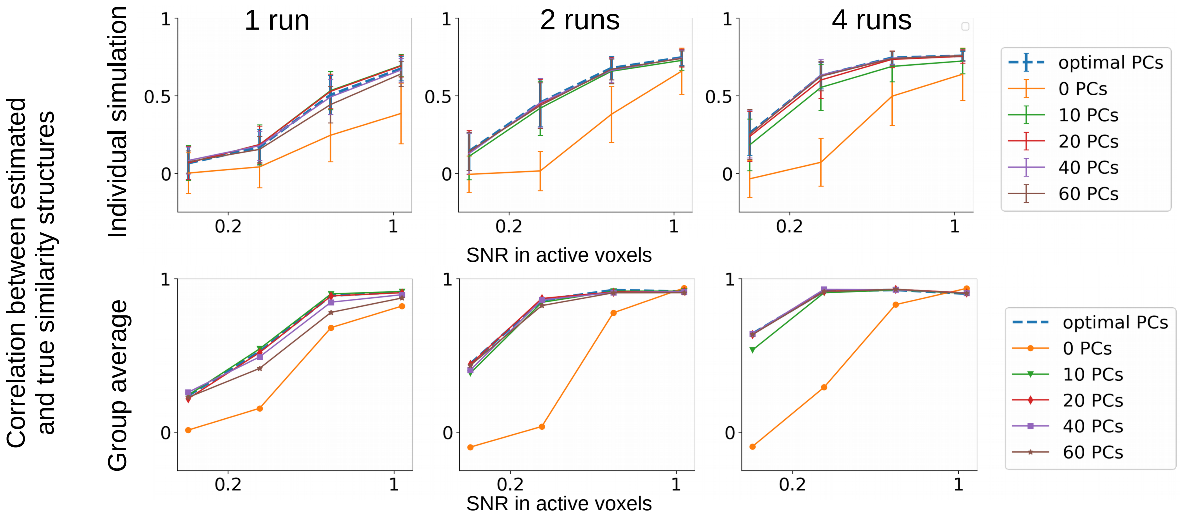

Figure 3: **The effect of the number of nuisance regressors on the performance of BRSA.** We used the same simulated data as Figure **3** in the main article, but varied the number of nuisance regressors used in BRSA model fitting. Top: average correlation (mean $\pm$ std) between the off-diagonal elements of the estimated and true similarity matrices, when using different number of nuisance regressors, across SNR levels (x-axis) and amounts of data (separate plots). "optimal PCs": the number of nuisance regressors is automatically determined. The numbers of PCs used to generate other curves are indicated in the legends Bottom: the correlation between the average estimated similarity matrix, depending on the number of nuisance regressors chosen.

(such as 40 or 60) hurts the performance. This likely would also happen if we included even larger number of nuisance regressors when fitting to larger amounts of data (2 and 4 runs). This comparison demonstrates that the number of nuisance regressors determined by the algorithm (5) is sufficient, and the small residual bias observed in BRSA result is not due to the number of nuisance regressors. Future investigation may help understand and further reduce this residual bias.

Part 7 Cross-validation with less stringent criterion

In Figure **5** of the main article we evaluated the rate of correctly accepting or correctly rejecting the full model in different scenarios, using paired t-test between the predictive log likelihoods of full and null model on left-out test data. When there is task-related signal in training data but not in test data, or when neither training nor test data contain task-related signal, t-test always correctly reject the full model, but it is also conservative when there is task-related signal in both training and test data. Here we display the rate of correctly accepting the full model when both data have task-related signal and correctly rejecting the full model in the other two corresponding scenarios, using a less stringent criterion of whether the difference between the predictive log likelihoods is larger than 0. Being able to accept the full model more frequently at low SNR (Figure **4A** of **S1** Materials), it also has small to medium false positive rate when either there is no task-related signal in the test data (Figure **4B** of **S1** Materials) or when neither data contain task-related signal (Figure **4C** of **S1** Materials)

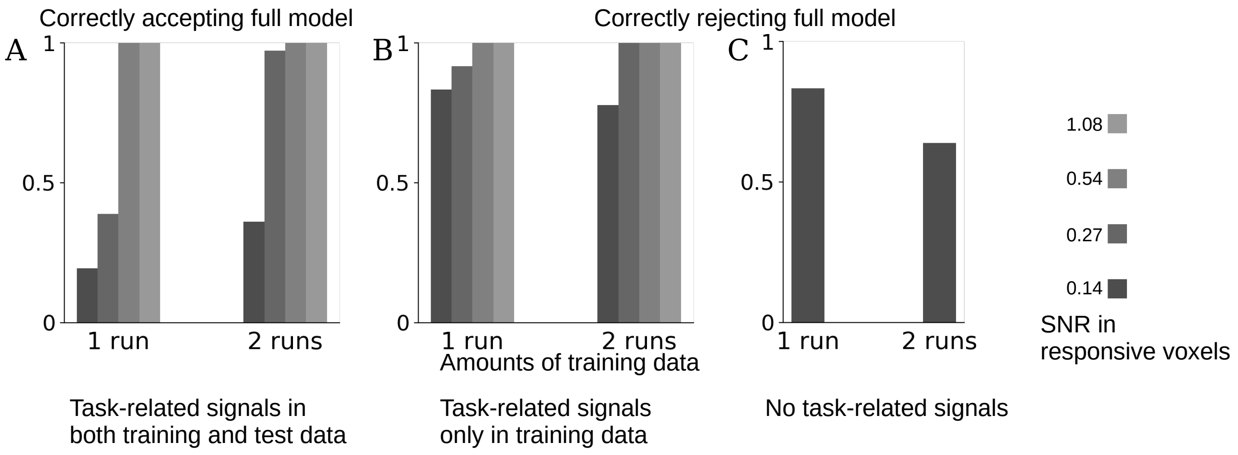

Figure 4: **Cross-validation performance using less stringent criterion** The same simulation was performed as in Figure **5** of the main text. But the criterion of deciding whether to accept the full model against the null model is based on whether the total predictive log likelihood of the full model on test data is higher than that of the null model. **(A)** The rate of correctly accepting the full model when there is consistent task-related signal in both training and test data. **(B)** The rate of correctly rejecting the full model when there is task-related signal in training, but not in test data. **(C)** The correct rejection rate when there is no task-related signal in either the training or test data. Shades of color indicate the SNR level in the task-active voxels, and different groups of bars correspond to different amounts of training data (1 or 2 runs)

References

[1] Broyden C. A new double-rank minimisation algorithm. Preliminary report. In: Notices of the American Mathematical Society. vol. 16. AMER MATHEMATICAL SOC 201 CHARLES ST, PROVIDENCE, RI 02940-2213; 1969. p. 670.

[2] Fletcher R. A new approach to variable metric algorithms. The computer journal. 1970;13(3):317–322.

[3] Goldfarb D. A family of variable-metric methods derived by variational means. Mathematics of computation. 1970;24(109):23–26.

[4] Shanno DF. Conditioning of quasi-Newton methods for function minimization. Mathematics of computation. 1970;24(111):647–656.

[5] Gavish M, Donoho DL. The optimal hard threshold for singular values is $4/\sqrt{3}$. IEEE Transactions on Information Theory. 2014;60(8):5040–5053.

[6] Bishop CM. Pattern recognition and machine learning. springer; 2006.

[7] Ephraim Y, Malah D, Juang BH. On the application of hidden Markov models for enhancing noisy speech. IEEE Transactions on Acoustics, Speech, and Signal Processing. 1989;37(12):1846–1856.
